# Ultrasound-Assisted Extraction of Curcumin Using a Deep Eutectic Solvent: Molecular Insights into the Extraction Mechanism

**DOI:** 10.3390/molecules31162818

**Published:** 2026-08-13

**Authors:** Ning Sun, Xinyu Guo, Enxi Li, Yan Zhou, Shuli Yin, Peizhe Cui

**Affiliations:** College of Chemical Engineering, Qingdao University of Science and Technology, Qingdao 266042, China; 19811789153@163.com (N.S.); 18698761301@163.com (X.G.); 18139376618@163.com (E.L.); 17860294335@163.com (Y.Z.); 04116@qust.edu.cn (S.Y.)

**Keywords:** curcumin, ultrasound-assisted extraction, liquid-solid extraction, deep eutectic solvents, quantum chemistry calculation

## Abstract

In this study, five levulinic acid-based deep eutectic solvents (DESs) were evaluated for the ultrasound-assisted extraction of curcuminoids from *Curcuma longa*. The relationship between DES composition, intermolecular interactions, and extraction performance was investigated by combining extraction experiments with spectroscopic characterization and quantum chemical calculations. The levulinic acid–ethylene glycol system at a 1:2 molar ratio exhibited the best performance, achieving an extraction yield of 99.9 mg·g^−1^. FTIR and ^1^H NMR analyses characterized the interactions within the DES, while SEM observations indicated structural disruption of the turmeric matrix after ultrasound treatment. Molecular analyses suggested that hydrogen bonding and van der Waals interactions contributed to the association between the DES and curcuminoids. These results provide molecular insights into the extraction process and a basis for designing greener solvent systems for curcuminoid extraction.

## 1. Introduction

With the increasing demand for natural food additives [1,2], curcuminoids have attracted considerable attention for their antioxidant, anti-inflammatory, anticancer, antidiabetic, antimutagenic, and antimicrobial activities [3,4]. *Curcuma longa* L. is a perennial herb widely cultivated in South and Southeast Asia and is commonly used as a spice, in traditional medicine, and as a natural colorant [5,6,7]. Its rhizomes are rich in curcuminoids, mainly curcumin (CUR), demethoxycurcumin (DMC), and bisdemethoxycurcumin (BDMC). These compounds belong to the diferuloylmethane family and coexist in keto and enol forms, with the enol form generally being more stable. CUR is the predominant and most bioactive component, accounting for approximately 75% of total curcuminoids, whereas DMC and BDMC account for about 10–20% and less than 5%, respectively, depending on geographical origin and cultivation conditions [8]. CUR can be obtained either by chemical synthesis or by extraction from natural sources. According to the Joint FAO/WHO Expert Committee on Food Additives, CUR used as a food additive should be of natural origin; therefore, efficient extraction from turmeric is particularly important [9].

At present, several extraction techniques have been proposed, including microwave-assisted extraction (MAE), ultrasound-assisted extraction (UAE), supercritical carbon dioxide extraction, and enzyme-assisted extraction [5,10,11,12,13,14,15,16]. Conventional extraction methods commonly employ organic solvents such as methanol, ethanol, isopropanol, and hexane [12,17,18,19]. However, they often require long processing times, consume large amounts of solvent, and involve additional recovery steps, thereby limiting their environmental and economic performance [20]. Although intensified techniques can improve extraction efficiency, microwave- and supercritical-fluid-based processes may require high energy input, strict operating conditions, or complex equipment, while enzyme-assisted extraction generally involves additional pretreatment steps [11,16,21,22,23]. In contrast, ultrasound-assisted extraction can enhance solvent penetration and mass transfer through cavitation and mechanical effects, while offering relatively low energy and solvent consumption [24,25]. Shirsath et al. [12] optimized the ultrasound-assisted extraction of CUR from turmeric and obtained a CUR content of 72 mg·g^−1^ using ethanol at 35 °C for 60 min and a solid-to-liquid ratio of 1:25. Wang et al. [26] further demonstrated that ultrasound-assisted extraction outperformed conventional solvent extraction, achieving a maximum extraction yield of 115.64 mg·g^−1^ at 25 °C for 60 min and a liquid-to-solid ratio of 21.58 mL·g^−1^. Nevertheless, these studies primarily focused on process optimization using conventional solvents, whereas the development of greener extraction media and the molecular interactions governing curcuminoid extraction remain insufficiently studied.

Deep Eutectic Solvents (DESs), first proposed by Abbott et al. [27], are generally formed through hydrogen-bond interactions between hydrogen bond acceptors (HBAs) and hydrogen bond donors (HBDs). Their melting points are markedly lower than those of the individual components. Although DESs are commonly described based on the HBA/HBD concept, some components may exhibit dual hydrogen-bonding functionalities depending on their molecular structures and interaction environments. Levulinic acid (ALCA), containing both carbonyl and carboxyl groups, can participate in DES formation through multiple hydrogen-bonding sites and should therefore be regarded as a multifunctional component rather than a simple HBA or HBD. Owing to their low volatility, simple preparation, and tunable physicochemical properties, DESs have been widely explored as alternatives to conventional organic solvents [26,28,29]. Fan et al. [30] showed that the selective extraction performance of DESs for alkaloids and anthraquinones was closely related to polarity, viscosity, pH, and hydrogen-bond interactions. Yan et al. [31] developed switchable DES systems responsive to CO_2_, pH, and temperature, enabling the extraction of compounds with different polarities while facilitating solvent recovery. Zuo et al. [32] summarized the application of NADESs in extracting flavonoids, phenolics, alkaloids, and pigments, while noting that toxicity and biodegradability still require further evaluation. Wang et al. [29] optimized a choline chloride–glycerol–citric acid DES for blueberry anthocyanin extraction and demonstrated that DES composition affected both extraction efficiency and selectivity. Compared with ionic liquids, DESs are generally easier to prepare and can often be obtained through a simple heating and mixing process [1,26].

In this work, five levulinic acid-based DESs were evaluated to elucidate the relationships among solvent composition, intermolecular interactions, and curcuminoid extraction performance. Unlike previous studies that focus mainly on solvent screening or process optimization, this study integrates FTIR, ^1^H NMR, extraction experiments, kinetic analysis, SEM, and quantum-chemical calculations into a unified framework. The levulinic acid–ethylene glycol system at a 1:2 molar ratio showed the best performance. Further molecular analyses indicated that hydrogen bonding and van der Waals interactions contributed to the extraction process. This study, therefore, establishes a structure–interaction–performance relationship for levulinic acid-based DESs and guides the design of greener solvents for curcuminoid extraction.

## 2. Results and Discussions

### 2.1. σ-Profile Analysis

To establish the relationship between DES molecular characteristics and extraction performance, the structural features and potential intermolecular interactions of ALCA-based DESs were first analyzed based on their σ-profiles. The σ-profile provides qualitative information on molecular polarity and hydrogen-bonding characteristics and has been widely used for solvent screening and phase-behavior prediction [33]. Mullins et al. [34] established a σ-profile database for common organic compounds, while Banerjee et al. [35] and Li et al. [36] demonstrated that the hydrogen-bonding characteristics of ionic or multicomponent systems could be evaluated from the profiles of their individual components. A similar component-based strategy was adopted in this study, and the molecular surface charge distributions were calculated according to previously reported procedures [30,37,38]. Using ±0.0082 e·Å^−2^ as the boundaries, the molecular surface was divided into hydrogen-bond-donor, nonpolar, and hydrogen-bond-acceptor regions [39].

**Figure 1:**
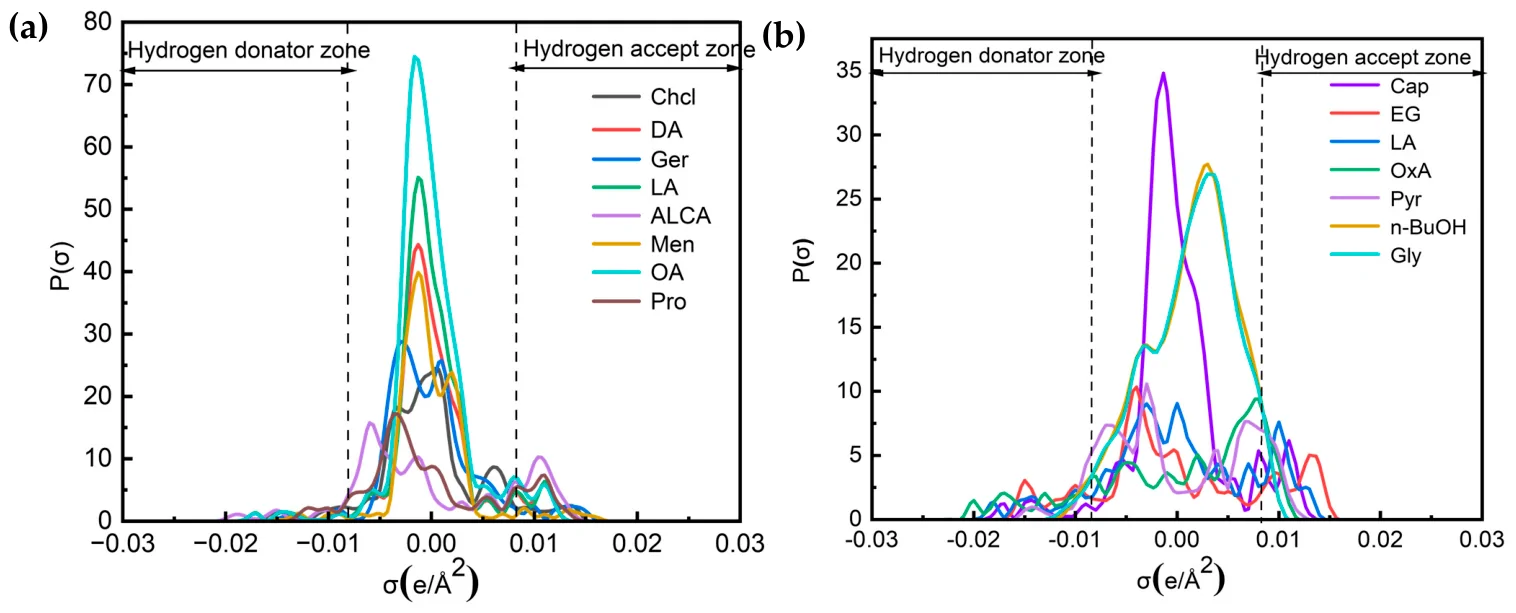
σ-Profiles of the investigated compounds: (**a**) ChCl, DA, Ger, LA, ALCA, Men, OA, and Pro; (**b**) Cap, EG, LA, OxA, Pyr, n-BuOH, and Gly.

As shown in Figure 1a, the eight candidate components exhibited distinct polar-surface distributions. ChCl and Pro showed pronounced hydrogen-bond-accepting characteristics, whereas ALCA displayed a broader distribution across both donor and acceptor regions. This behavior is associated with the carbonyl-, carboxyl-, and hydroxyl-related interaction sites of ALCA, suggesting that it can participate in diverse intermolecular interactions. Figure 1b further shows that Pyr, OxA, EG, LA, n-BuOH, Gly, and Cap possess different polar and nonpolar surface characteristics because of their structural differences.

Based on its broad polar distribution and multiple potential interaction sites, ALCA was selected as the principal component for preparing the candidate DESs. However, because ALCA contains both hydrogen-bond-donating and hydrogen-bond-accepting functionalities, its role should not be considered exclusively that of an HBA. Moreover, the σ-profile was used only for preliminary screening, whereas the final solvent selection was based on experimental extraction performance because viscosity, molar ratio, component compatibility, and solvent–solute interactions may also affect extraction.

### 2.2. Selection and Characterization of DESs

The FTIR spectra of the individual components (ALCA, EG, Pyr, and LA) and the corresponding DESs (ALCA/EG, ALCA/Pyr, and ALCA/LA) are shown in Figure 1a,b. No new absorption bands appeared after DES formation, indicating that no covalent reactions occurred between ALCA and the hydrogen-bond-donor components, which is consistent with previous reports on DES formation through noncovalent interactions [3]. After DES formation, the characteristic bands associated with C=O, –COOH, and –OH groups exhibited slight shifts, suggesting that the local chemical environments of these functional groups were altered due to intermolecular interactions between ALCA and hydrogen-bond donors [40]. Similar spectral variations have been reported in DES systems and are generally associated with changes in hydrogen-bonding environments [41]. Therefore, the FTIR results provide supportive evidence for the formation of intermolecular interaction networks within DESs rather than direct proof of hydrogen bonding. ^1^H NMR and theoretical calculations were used to investigate the detailed interaction characteristics further.

**Figure 2:**
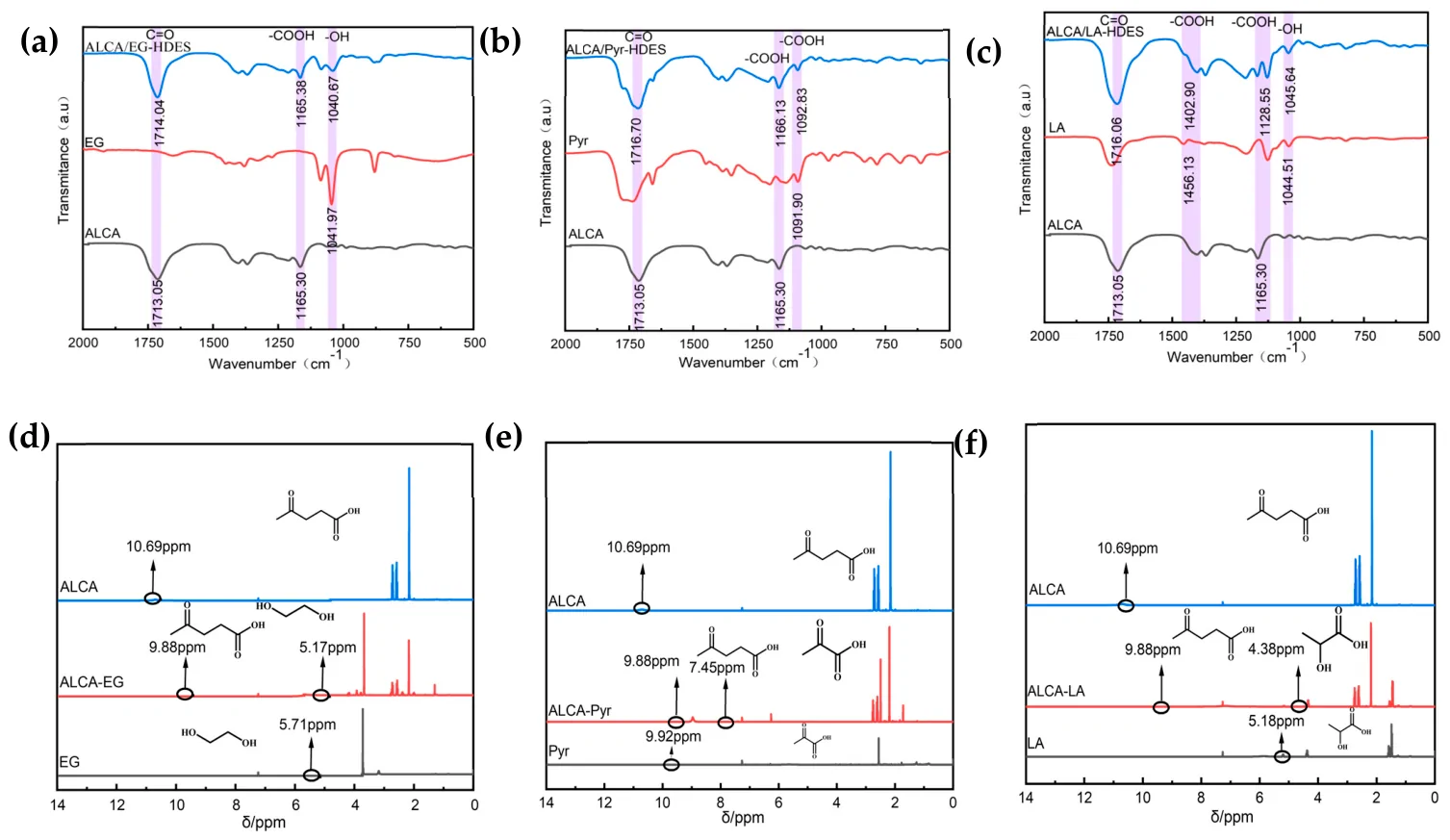
FTIR and ^1^H NMR spectra of levulinic acid-based deep eutectic solvents and their individual components: (**a**–**c**) FTIR spectra of ALCA/EG, ALCA/Pyr, and ALCA/LA systems; (**d**–**f**) ^1^H NMR spectra of ALCA/EG, ALCA/Pyr, and ALCA/LA systems.

Figure 2d–f shows the ^1^H NMR spectra of ALCA, EG, Pyr, LA, and the corresponding DESs (complete spectra are provided in Figures S3 and S4). The characteristic proton signals of both ALCA and hydrogen-bond donor components were retained after DES formation, and no new signals appeared, indicating the absence of covalent reactions during preparation. Compared with the individual components, the hydroxyl proton signals of ALCA and EG shifted from 10.69 and 5.71 ppm to 9.88 and 5.17 ppm, respectively, with signal broadening. These changes indicate variations in the local proton environments arising from intermolecular interactions among DES components. Similar chemical shift changes have been reported in carboxylic acid-based DES systems and were attributed to alterations in hydrogen-bonding environments [42]. Therefore, the ^1^H NMR results, together with FTIR and theoretical analysis results, support the formation of noncovalent interaction networks within ALCA-based DESs.

### 2.3. Selection of DESs

Based on the predicted intermolecular interaction characteristics, FTIR and ^1^H NMR analyses were further performed to experimentally verify the formation of noncovalent interaction networks within the DES systems. As summarized in Table 1, the curcuminoid extraction yields obtained using different green solvent systems ranged from 11.9 to 99.9 mg·g^−1^. Previous studies have mainly focused on choline chloride-based DESs for curcuminoid extraction [1,5,7,36]. However, the influence of alternative hydrogen-bonding components on extraction performance remains insufficiently explored. Therefore, this study investigated the effects of different ALCA-based DES systems prepared with various hydrogen-bonding components under ultrasound-assisted extraction conditions (100 W, 35 °C, 55 min). The extraction performance was strongly dependent on DES composition, and the ALCA/EG system (molar ratio 1:2) exhibited the highest curcuminoid yield of 99.9 mg·g^−1^, exceeding that of conventional 80% ethanol extraction. In contrast, the ChCl/Pyr system showed a much lower yield (11.9 mg·g^−1^), highlighting the critical role of DES molecular structure in regulating extraction performance. Previous studies have demonstrated that choline chloride-based DESs and natural deep eutectic solvents can effectively extract bioactive compounds from plant matrices due to their tunable polarity and hydrogen-bonding ability [42,43,44]. However, extraction efficiencies vary considerably depending on DES composition, target compounds, and extraction conditions [45]. Compared with previously reported DES-based extraction systems, the ALCA/EG system exhibited competitive extraction performance, which may be attributed to its suitable polarity and multiple hydrogen-bonding sites.

### 2.4. Optimization of Extraction Experimental Conditions

Many factors may affect the yield of curcumin extract. To determine the correlation between extraction conditions and target components, the effects of ultrasound power, molar ratio of hydrogen bond acceptor to hydrogen bond donor, liquid-solid ratio, and ultrasound time on the equilibrium extraction rate of curcumin were evaluated using single-factor experiments.

#### 2.4.1. Ultrasound Power

**Figure 3:**
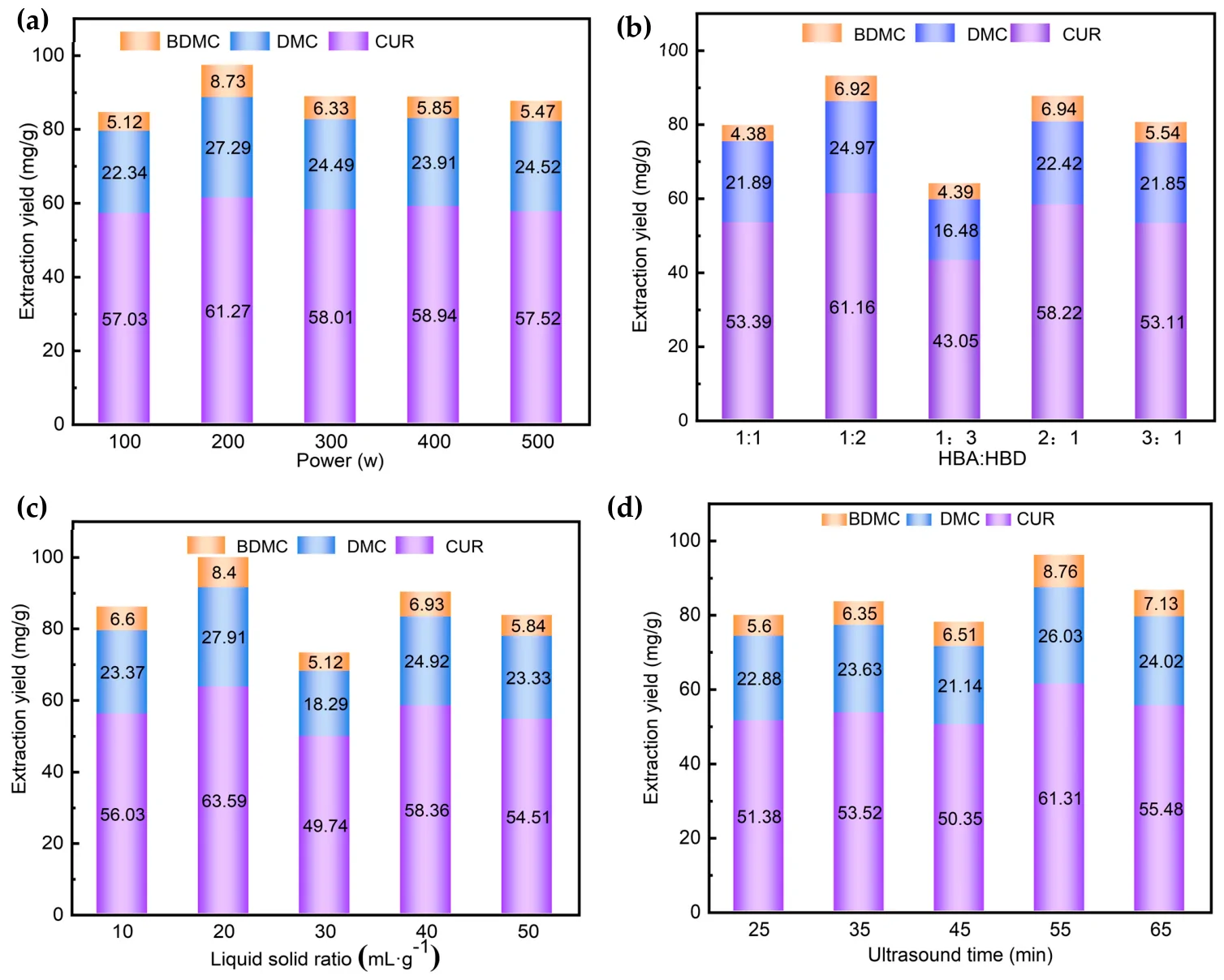
Extraction of CURs under different extraction conditions; the conditions of each subgraph are as follows: (**a**) ultrasound power (w; experimental conditions: liquid-solid ratio 20 mL·g^−1^, ultrasound time 30 min, extraction temperature 30 °C); (**b**) molar ratio of hydrogen bond acceptor and hydrogen bond donor (experimental conditions: liquid-solid ratio 20 mL·g^−1^, extraction temperature 30 °C, ultrasound time 30 min, ultrasound power 80 (w); (**c**) liquid-solid ratio (experimental conditions: extraction temperature 30 °C, ultrasound time 30 min, ultrasound power 80 w); (**d**) ultrasound time (min; experimental conditions: liquid-solid ratio 20 mL·g^−1^, extraction temperature 30 °C, ultrasound power 80 w).

The effect of ultrasound power on the extraction of three curcuminoids is shown in Figure 3a. The highest extraction yield of curcuminoids was 97.26 mg·g^−1^. At 200 W, the extracted amounts of DMC and CUR reached their highest values. BDMC extraction was relatively high at a lower power (100 W). When the power exceeded 200 W, the extracted amounts of the three compounds decreased to varying degrees, indicating that excessive ultrasound power may degrade the target component due to excessive cavitation or local temperature rise. Therefore, the appropriate ultrasound power improves the extraction efficiency for curcuminoids.

#### 2.4.2. Different Molar Ratios of HBA to HBD

The molar ratio of HBA to HBD had a significant effect on the total extraction of curcuminoids. (Figure 3b). When HBA:HBD was 1:2, the total curcumin content reached the highest value; compared with the ratios 1:1, 1:3, 2:1, and 3:1, the extraction efficiency under this condition showed varying degrees of improvement. The results showed that the appropriate HBA:HBD ratio (1:2) improves the overall dissolution effect of curcuminoids. In contrast, excessive receptor or donor amounts would lead to a decrease in the total extracted amount.

#### 2.4.3. Liquid-Solid Ratio

The liquid-solid ratio had a significant effect on the total amount of curcuminoids extracted. Figure 3c shows that a liquid-solid ratio of 20 mL·g^−1^ is sufficient to fully contact the solid surface and dissolve curcuminoids, thereby achieving the best extraction efficiency. As the liquid-solid ratio increased, the total curcumin content gradually decreased. The above results showed that the optimal liquid-solid ratio (20 mL·g^−1^) improved the overall dissolution efficiency of curcuminoids. The high liquid-solid ratio decreased the trichoflavin concentration in the solution, reduced the diffusion driving force in the extraction process, and reduced the extracted amount [46]. In addition, a liquid-solid ratio that is too high will increase operating costs.

#### 2.4.4. Extraction Residence Time

Ultrasound time had a significant effect on the total amount of curcuminoids extracted. As shown in Figure 3d, the total curcumin content increased gradually with the extension of ultrasound time from 25 min to 55 min, and reached 96.1 mg·g^−1^ at 55 min. This is attributed to the cavitation and mechanical effects of ultrasound, which significantly enhance the initial rupture of the cell wall, thereby promoting the release of the cytosol. When the ultrasound time was extended to 65 min, the total curcumin content decreased significantly, and prolonged ultrasonic time may lead to curcumin degradation or oxidation [5].

### 2.5. Scanning Electron Microscopy (SEM) Characterization

SEM analysis was performed to investigate the morphological changes of turmeric powder before extraction, after HDES extraction without ultrasound treatment, and after ultrasound-assisted HDES extraction [47]. All samples were pretreated with vacuum gold sputtering for 60 s and subsequently examined using a scanning electron microscope at 10,000× magnification with an accelerating voltage of 25 kV.

**Figure 4:**
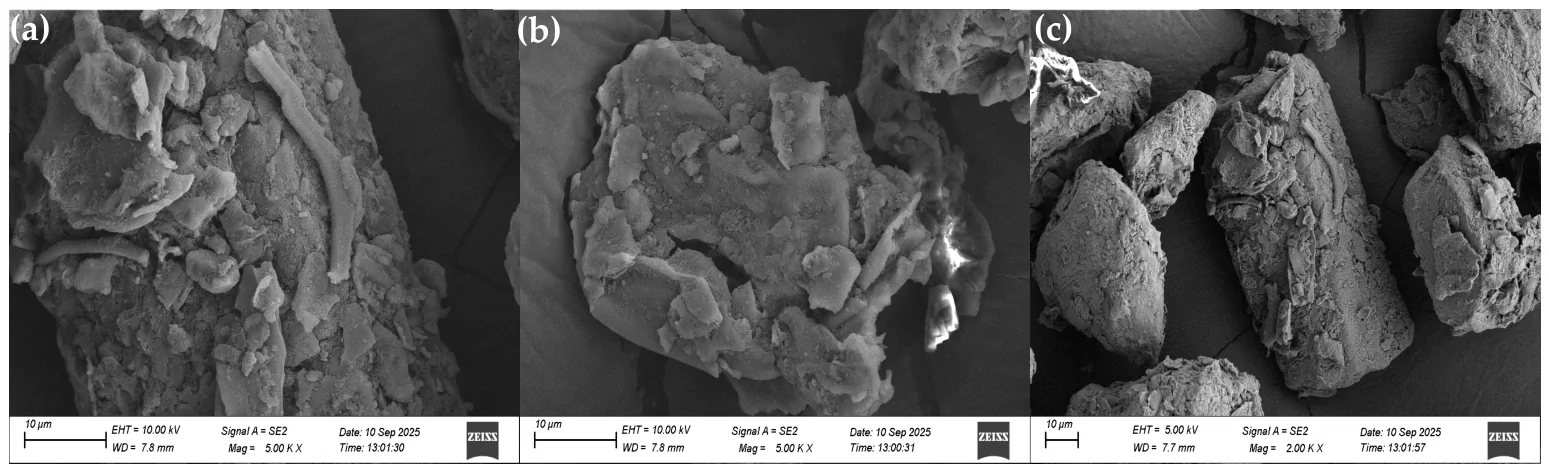
SEM images of turmeric root powder: (**a**) before extraction; (**b**) after HDES extraction without ultrasound treatment; (**c**) after ultrasound-assisted HDES extraction.

As shown in Figure 4a, untreated turmeric powder exhibited a relatively intact structure with a rough surface morphology. After HDES extraction without ultrasound treatment (Figure 4b), the particle structure was largely preserved, although slight surface cracks were observed. In contrast, ultrasound-assisted HDES extraction resulted in more pronounced morphological changes, including increased surface fragmentation, cracks, and thinner particle structures (Figure 4c). These observations indicate that ultrasound treatment induces structural disruption of turmeric particles, which is consistent with previous studies reporting that ultrasound-induced cavitation can modify plant matrices and improve solvent accessibility during extraction [48,49,50].

It should be noted that SEM characterization primarily provides morphological evidence and cannot directly verify mass-transfer enhancement or extraction mechanisms. Therefore, the improved extraction performance is attributed to the synergistic effects of ultrasound-induced cavitation, enhanced solvent accessibility, and favorable DES–curcuminoid interactions.

### 2.6. Analysis of Extraction Mechanism

To obtain molecular-level insights into the interactions responsible for the observed extraction behavior, quantum chemical calculations were subsequently performed. ESP plays an important role in the study of molecular electrostatic interactions and the prediction of molecular properties, and is widely used because of its vivid and intuitive advantages [51]. In order to more clearly observe which surface van der Waals (vdW) forces penetrate between molecules and discuss the strength of the interaction, we used Multiwf 3.8 and VMD 1.9.4 software to draw the ESP penetration map [52].

#### 2.6.1. Interaction Energy

As shown in Table 2, the interaction energies of EG, LA, Pyr, Cap, and OxA with ALCA are −0.0323, −0.0260, −0.0128, −0.0116, and −0.0159, respectively. The interaction energies between EG and ALCA and between ALCA/EG and CUR-DMC-BDMC are the largest. Table 2 shows that the interaction energy between ALCA/EG and CUR-DMC-BDMC is the strongest.

**Figure 5:**
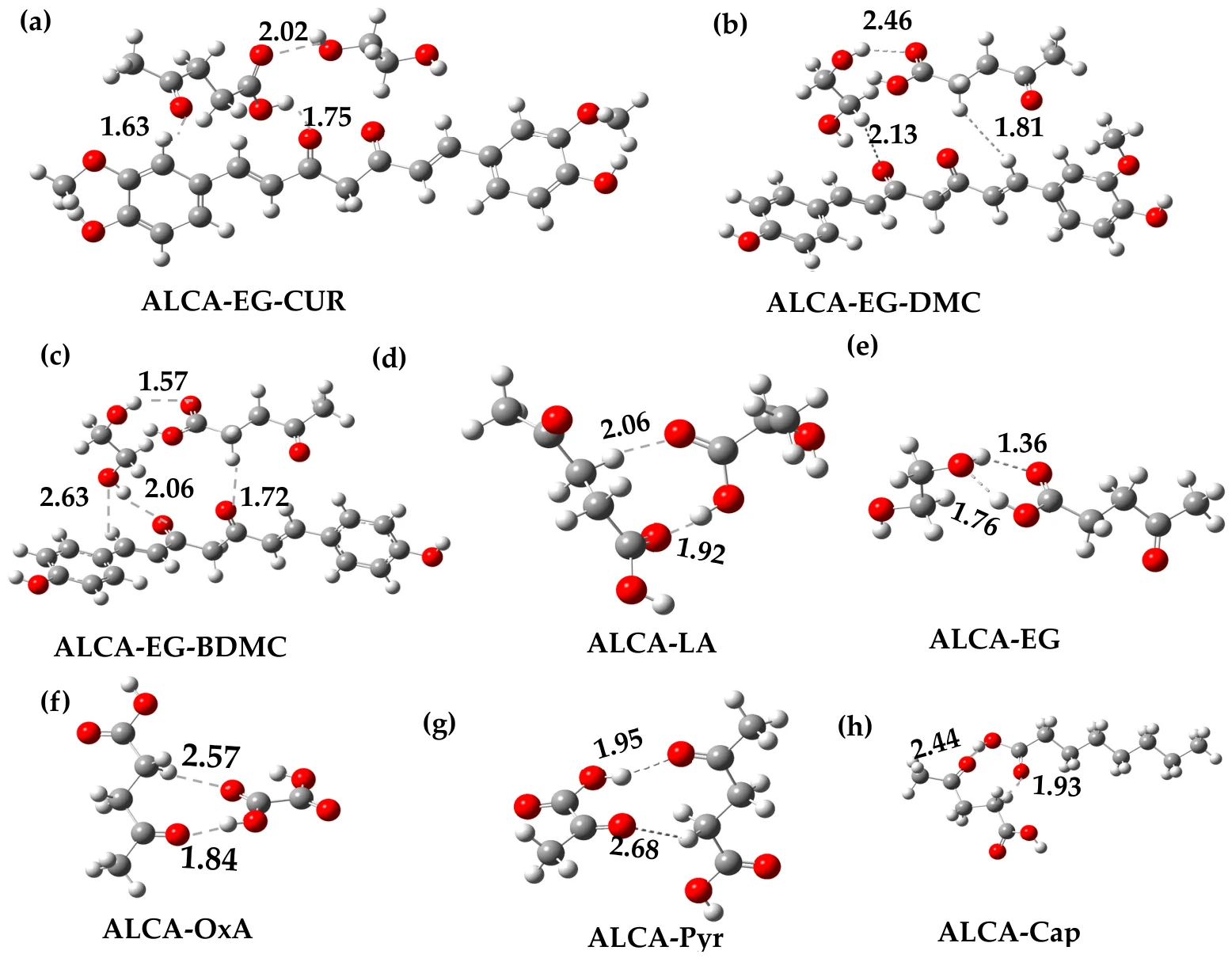
Molecular interactions between levulinic acid-based DESs and curcuminoids: (**a**) ALCA/EG-CUR; (**b**) ALCA/EG-DMC; (**c**) ALCA/EG-BDMC; (**d**) ALCA/LA; (**e**) ALCA/EG; (**f**) ALCA/OxA; (**g**) ALCA/Pyr; (**h**) ALCA/Cap.

Figure 5 shows the bonding potential of the DES to CURs. The relationship between DES and different substances is shown in Figure 5. As the bond length decreases, the bond energy increases, allowing the molecule to form a stable structure. Figure 5a–c show the bonding relationship between ALCA/EG and CURs. In the interaction between ALCA/EG and CURs, ALCA mainly forms hydrogen bonds with substances. The O atom of ALCA can form a force with the H atom in curcuminoids. The bond length of ALCA and CUR is 1.63, the bond length of ALCA and DMC is 1.81, and the bond length of ALCA and BDMC is 1.72. ALCA is more likely to form a stable structure with CURs. ALCA plays a significant role in forming interactions with CURs.

#### 2.6.2. Van der Waals Electrostatic Potential Analysis of Molecular Surface

**Figure 6:**
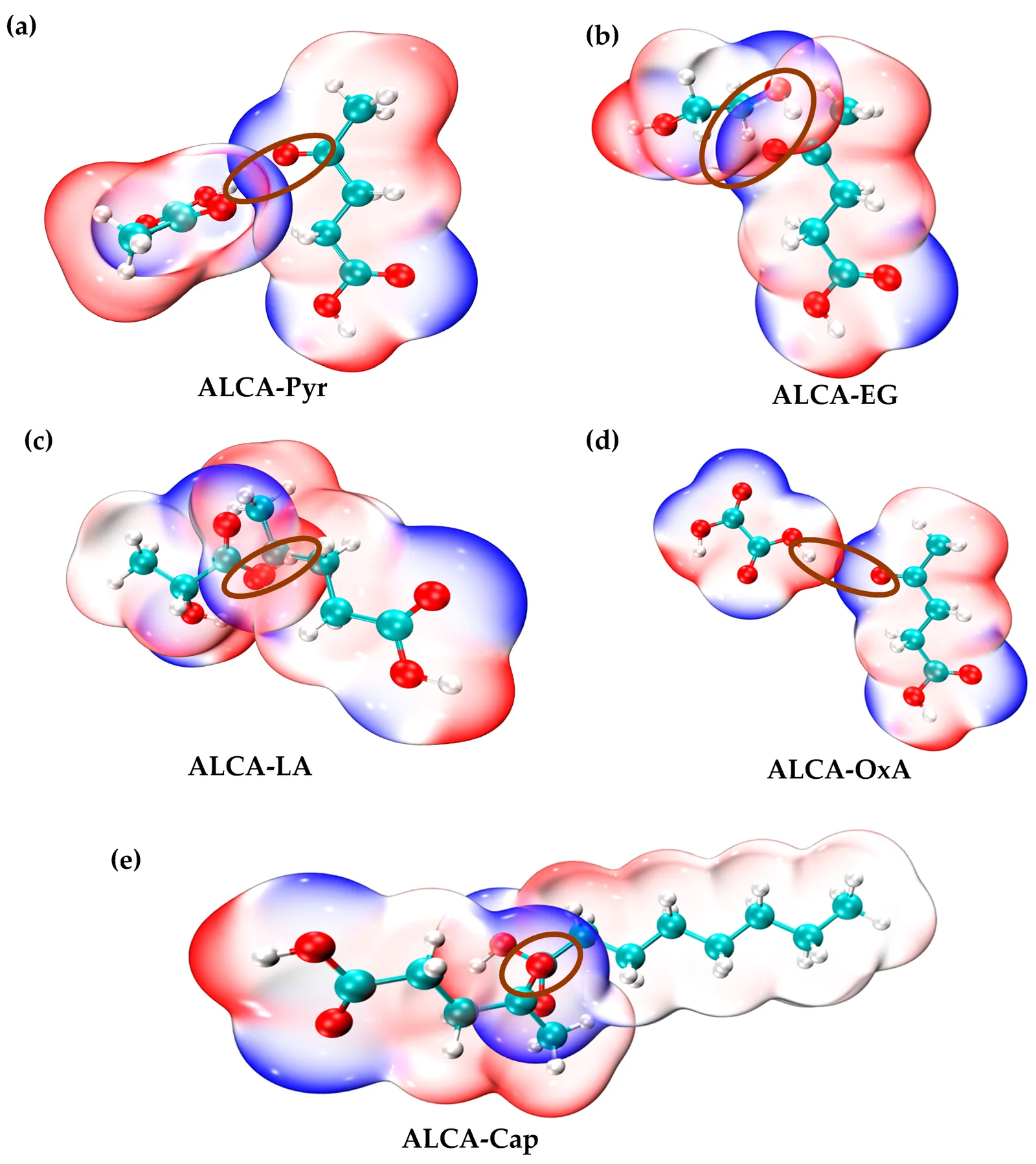
Analysis diagrams of van der Waals electrostatic potential for the DES complexes: (**a**) ALCA–Pyr; (**b**) ALCA–EG; (**c**) ALCA–LA; (**d**) ALCA–OxA; and (**e**) ALCA–Cap. (red: positive potential; blue: negative potential).

Figure 6 presents the molecular electrostatic potential (ESP) distributions of ALCA with different hydrogen-bonding components (EG, Pyr, OxA, LA, and Cap). The oxygen atoms in ALCA exhibit pronounced negative potential regions. In contrast, the hydrogen atoms of the hydroxyl and carboxyl groups show positive potentials, indicating sites for electrostatic and hydrogen-bond interactions. Among the investigated systems, ALCA/EG exhibited the most complementary potential distribution, with extensive overlap between electron-rich and electron-deficient regions, suggesting stronger intermolecular interactions, which is consistent with the σ-profile analysis.

For the ALCA/Pyr, ALCA/OxA, and ALCA/Cap systems, the interaction mainly involved the electron-deficient hydrogen atoms of carboxyl groups and the electron-rich oxygen atoms of ALCA. In contrast, the presence of hydroxyl groups in LA resulted in a different interaction pattern, where competing hydrogen-bonding sites reduced the electrostatic complementarity with ALCA. Overall, the ESP analysis reveals the potential interaction sites and polarity matching between ALCA and different components, providing molecular-level insights into DES formation.

ESP analysis of interaction binding sites can provide a theoretical basis for the binding mode of DES based on ALCA, helping to predict its extraction potential. A stronger intermolecular interaction does not necessarily result in better extraction performance, because the accessibility of complementary electrostatic sites and the competition between DES–DES and DES–solute interactions should also be considered [53,54]. The ESP permeation diagrams of ALCA and various HBD molecules with CUR, DMC, and BDMC are shown in Supplementary Materials Figures S5–S7. There is a strong positive potential near the phenolic hydroxyl H in CUR, and a strong negative potential near the carbonyl O, which can form a deep potential penetration zone with ALCA (Supplementary Materials Figure S5a), indicating that there is a strong interaction between them. CUR and HBD molecules can also form a certain interaction; however, the penetration depth is not as deep as that of ALCA, indicating that ALCA plays a leading role in the extraction process. The DMC and BDMC, as well as CUR, molecules have similar chemical structures. Both the positive potential near the phenolic hydroxyl H and the negative potential near the carbonyl O penetrate ALCA, resulting in a strong interaction.

#### 2.6.3. IGMH Analysis

The independent gradient model based on Hirshfeld partition (IGMH) was employed to visualize the nature and spatial distribution of noncovalent interactions between ALCA/EG and CUR, DMC, and BDMC. In the mapped isosurfaces, blue regions represent relatively strong attractive interactions, particularly hydrogen bonding; green regions indicate dispersion-dominated van der Waals interactions; and red regions correspond to steric repulsion [55,56].

**Figure 7:**
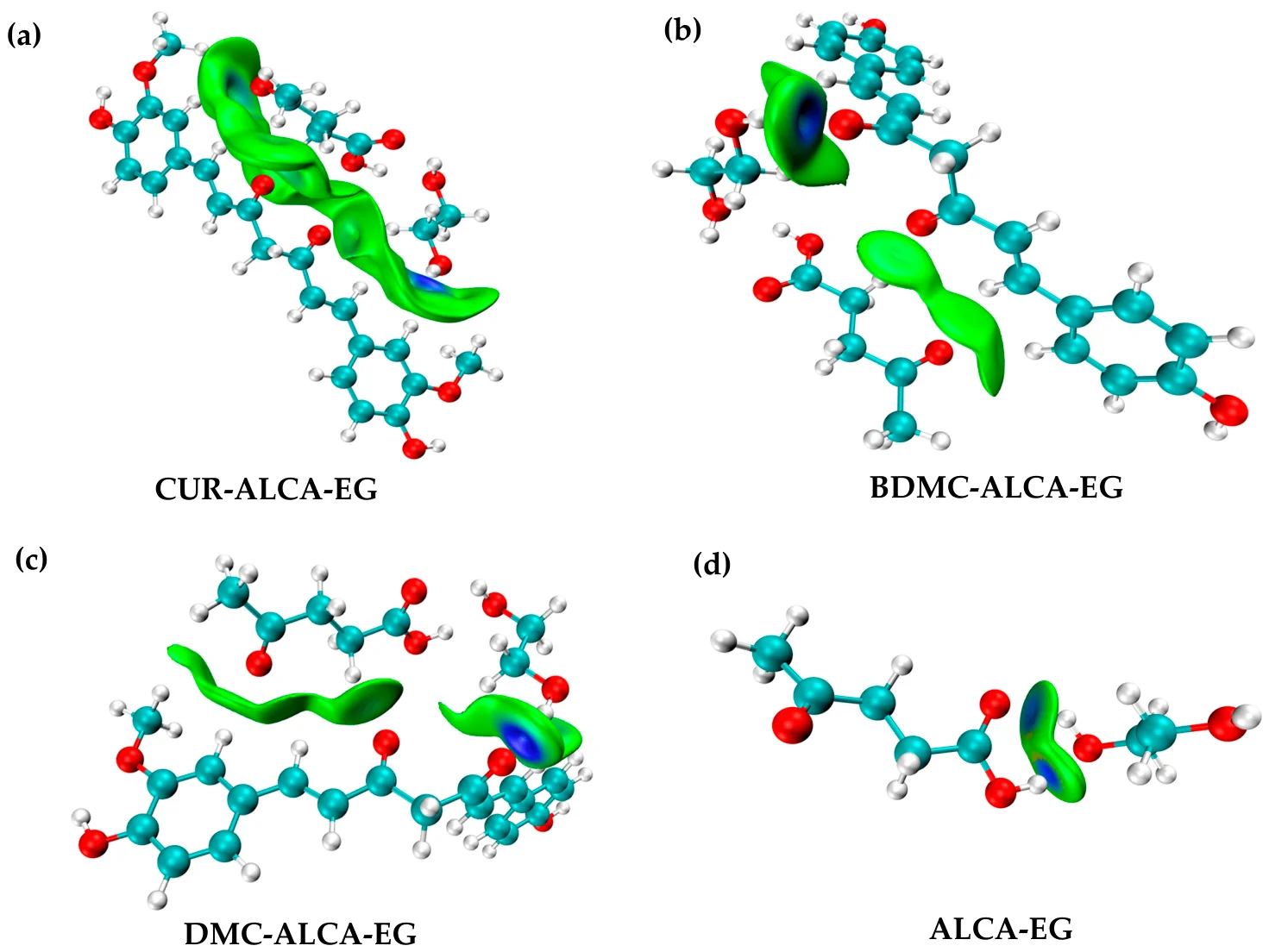
IGMH between different components with CUR-DMC-BDMC: (**a**) CUR-ALCA/EG; (**b**) BDMC-ALCA/EG; (**c**) DMC-ALCA/EG; (**d**) ALCA/EG. (Blue indicates the presence of hydrogen bonds between molecules, green indicates the presence of van der Waals interactions between molecules, and red indicates steric hindrance. The range of electron density values is: −0.04–+0.04).

As shown in Figure 7a–c, extensive green isosurfaces are distributed between ALCA/EG and the molecular frameworks of the three curcuminoids, indicating that dispersion interactions contribute substantially to their association. Localized blue or blue–green regions are mainly observed between the phenolic hydroxyl or carbonyl groups of the curcuminoids and the polar groups of ALCA and EG. These features indicate that hydrogen bonding and van der Waals interactions coexist at different molecular sites and jointly stabilize the DES–curcuminoid complexes.

The IGMH map of ALCA/EG also exhibits attractive regions between the hydroxyl- and carbonyl-containing sites of the two DES components, supporting the formation of an internal noncovalent interaction network. Meanwhile, both ALCA and EG provide accessible polar sites for association with the phenolic hydroxyl and carbonyl groups of the curcuminoids. Their contributions should therefore be considered cooperative, although their relative contributions may differ.

As shown in Figure S9, the ALCA/EG–BDMC system exhibits more extensive attractive regions than the other investigated ALCA-based DES–BDMC systems. Further comparison of ALCA–BDMC and EG–BDMC suggests that ALCA contributes more strongly to the localized attractive interactions, whereas EG provides additional complementary interaction sites. Separately, the calculated interaction energies of ALCA/EG with CUR, DMC, and BDMC were −0.0415, −0.0267, and −0.0112 a.u., respectively, with the strongest predicted association occurring with CUR. Overall, the IGMH and interaction-energy results demonstrate that hydrogen bonding and dispersion interactions jointly govern the association of ALCA/EG with curcuminoids, with ALCA and EG exhibiting cooperative but non-equivalent contributions.

#### 2.6.4. Interaction Area Index Analysis

**Figure 8:**
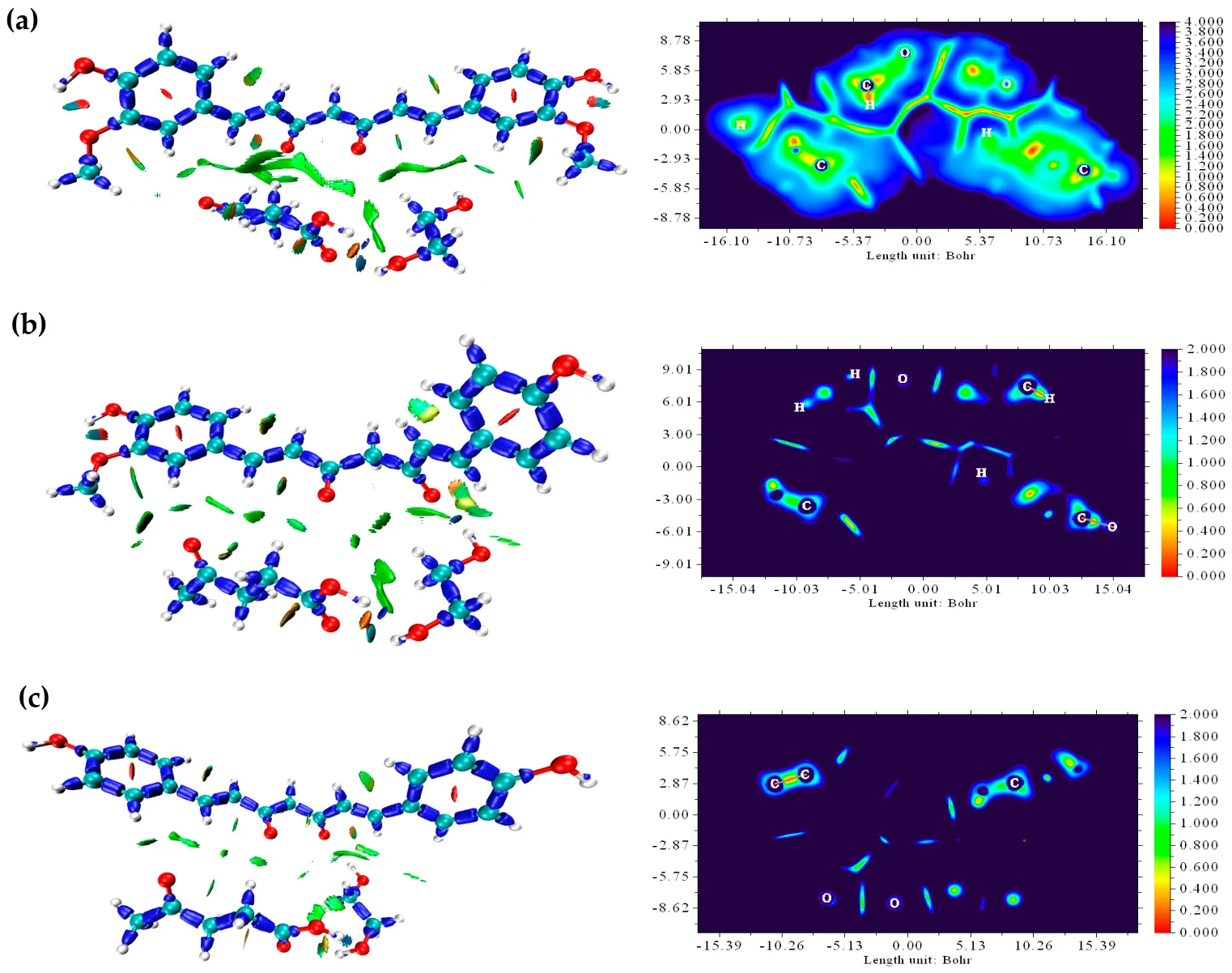
(**a**) IRI contour map and IRI colored map for CUR-DESs1; (**b**) DMC-DESs1; (**c**) BDMC-DESs1.

The Independent Gradient Model based on Hirshfeld partition (IRI) was further employed to visualize the noncovalent interactions between DES components and curcuminoids. In the IRI map, blue, green, and red regions represent strong attractive interactions, weak van der Waals interactions, and steric repulsion, respectively [57]. As shown in Figure 8, various noncovalent interactions, including hydrogen bonding and van der Waals interactions, were observed between DES components and curcuminoids. The interaction regions between CUR and DESs exhibited more pronounced attractive characteristics than those between DMC and BDMC, suggesting stronger molecular associations between CUR and DESs. These results provide molecular-level insights into potential selective interactions between DESs and various curcuminoids.

Since the interaction patterns of the investigated DESs with curcuminoids were similar, the IRI analysis of DES1 (ALCA/EG) was selected as a representative example (Figure 8). The results revealed that CUR, DMC, and BDMC mainly interacted with DES1 through hydrogen bonding and van der Waals interactions. Among them, CUR exhibited more pronounced attractive interaction regions with DES1, involving interactions with both ALCA and EG components, whereas DMC and BDMC showed relatively weaker interactions. The IRI analyses of individual DES components with different curcuminoids (Supplementary Materials Figures S10–S12) further confirmed the presence of noncovalent interactions after DES formation. These results suggest that the enhanced interaction between DES components and curcuminoids, particularly CUR, may contribute to the observed differences in extraction performance. Collectively, the above results establish a structure–interaction–performance relationship for ALCA-based DESs, where molecular interactions, solvent properties, and extraction behavior are interconnected. The combined experimental and computational analyses provide complementary evidence for understanding the factors affecting curcuminoid extraction.

## 3. Experimental and Methods

### 3.1. Materials and Solvents

The dried ginger yellow powder of the *C. longa* species was purchased from Leshan, Sichuan, and 98% pure CUR standard, DMC (>98%), and BDMC (>98%) were provided by Shanghai McLean Biochemical Technology Co., Ltd., Shanghai, China. Oxalic acid (OxA), ethylene glycol (EG), pyruvate (Pyr), lactic acid (LA), levulinic acid (ALCA), caprylic acid (Cap), glycerol (Gly) and n-butanol (n-BuOH) were purchased from Shanghai McLean Biochemical Technology Co., Ltd., Shanghai, China. Chromatographic-grade methanol and HPLC-grade acetonitrile and phosphoric acid were purchased from Shanghai Aladdin Biochemical Technology Co., Ltd., Shanghai, China.; the purity of these substances is 99%. HPLC-grade deionized water purified by a Millipore Milli Q 50 system was used (Merck, Darmstadt, Germany).

### 3.2. Instruments

An Agilent 1220 high-performance liquid chromatography system (Agilent Technologies, Santa Clara, CA, USA) was used for curcuminoid analysis. Ultrasound-assisted extraction was performed using an ultrasonic bath (KQ-300DE, Kunshan Ultrasound Instrument Co., Ltd., Shanghai, China) operating at a fixed frequency of 40 kHz and a rated power of 400 W. The applied ultrasonic power was adjustable according to the experimental design. No programmed on/off pulse cycle was applied; therefore, the instrument was operated in continuous ultrasound mode. A microanalytical balance (Shanghai Tianmei Balance Instrument Co., Ltd., Shanghai, China), 1 mL syringes, and 0.45 μm disposable membrane filters were also used. The morphology of turmeric powder before and after extraction was observed using scanning electron microscopy (SEM, ZEISS EVO MA15, Carl Zeiss Microscopy GmbH, Jena, Germany).

### 3.3. Extraction Experiments

A 1.0 g portion of turmeric powder was added to a 50 mL round-bottom flask containing 20 mL of DES. The flask was placed in the ultrasonic bath and subjected to ultrasound-assisted extraction at 40 kHz. The ultrasound bath had a rated power of 400 W, while the applied ultrasonic power was adjusted according to the single-factor experimental design. Unless otherwise specified, the extraction was conducted at 35 °C and 200 W for 55 min in continuous ultrasound mode.

After extraction, the mixture was centrifuged at 8000 rpm for 5 min to separate the residual turmeric powder. The supernatant was collected and appropriately diluted with HPLC-grade methanol. The diluted solution was thoroughly mixed, filtered through a 0.45 μm membrane, and analyzed according to the HPLC procedure described in Section 2.5. All extraction experiments were performed in triplicate.

### 3.4. Kinetic Model

In this study, the semi-empirical kinetic model developed by Peleg was used to determine the initial extraction rate, equilibrium concentration and extraction rate constant [58]. Due to the similar shape of the adsorption curve, it has been reported that the Peleg model is widely used to explain the modified extraction curve of bioactive molecules from natural sources. Karacabey et al. [59] and Shirsath et al. [12] applied the Peleg model to explain ultrasound-assisted extraction of natural ingredients. Using the model equation to extract the dynamics of molecules can be given as follows:

(1) $$M_{t}=M_{0}+\frac{t}{K_{1}+K_{2}t}$$

where *M*_t_ is the concentration of curcumin at time t (mg curcuminoids/g turmeric powder), *K*_1_ is Peleg’s rate constant (min. g/mg) and *K*_2_ is Peleg’s capacity constant (g/mg). *M*_0_ is the initial concentration of curcumin, which is zero as fresh solvent is used. The extraction typically occurs in two stages with the first order trend in the initial stage and zero order trend in the later. The modified Peleg’s equation, which represents the concentration of target solute (curcumin) in extraction solvent vs. time, can be written as follows:

(2) $$M_{t}=\frac{t}{K_{1}+K_{2}t}$$

The graph of 1/*M*_t_ vs. 1/t can be plotted to calculate *K*_1_ (Peleg’s rate constant) and *K*_2_ (Peleg’s capacity constant) values from the slope and intercept, respectively. *M*_t_ was also subsequently calculated using Equation (2) at different times to check the fit of the model.

### 3.5. HPLC Analysis

Individual stock solutions of curcumin (CUR), demethoxycurcumin (DMC), and bisdemethoxycurcumin (BDMC) were prepared in HPLC-grade methanol and further diluted with methanol to obtain a series of standard solutions within the concentration range of 2–12 μg·mL^−1^. Calibration curves were constructed by plotting the chromatographic band area against the corresponding analyte concentration (Supplementary Materials Figure S1).

After extraction, the samples were appropriately diluted with HPLC-grade methanol to ensure that the concentrations of the three curcuminoids were within the calibration range. The diluted solutions were thoroughly mixed and filtered through a 0.45 μm membrane before HPLC analysis.

Curcuminoids were analyzed using an Agilent 1220 HPLC system (Agilent Technologies, Santa Clara, CA, USA) equipped with an InertSustain C18 column (250 mm × 4.6 mm, 5 μm). The mobile phase consisted of an aqueous 0.5% phosphoric acid solution and acetonitrile at a volume ratio of 40:60 under isocratic elution. The flow rate was 0.9 mL·min^−1^, the column temperature was 30 °C, the detection wavelength was 425 nm, and the injection volume was 20 μL. CUR, DMC, and BDMC were identified by comparing their retention times with those of authentic standards and quantified using their respective calibration curves (Supplementary Materials Figure S2).

### 3.6. Quantum Chemistry Calculation

Initial molecular structures were constructed using GaussView 5.0. Candidate configurations of the DES components and DES–curcuminoid complexes were generated using the Genmer module of Molclus (version 1.9.9.9) and preliminarily optimized using MOPAC 23.2.5. The resulting structures were ranked according to their energies, and geometrically similar or duplicated configurations were removed. Representative low-energy structures were subsequently optimized using Gaussian 09W at the B3LYP/6-311G(d,p) level [60,61]. The SCF convergence threshold was set to 1.0 × 10^−8^ a.u. Geometry optimization was considered converged when the maximum force, root-mean-square force, maximum displacement, and root-mean-square displacement were below 4.5 × 10^−4^, 3.0 × 10^−4^, 1.8 × 10^−3^, and 1.2 × 10^−3^ a.u., respectively. No implicit solvation model was applied; therefore, the calculated energies were used for relative comparison of the molecular clusters rather than as absolute solution-phase thermodynamic quantities. The B3LYP/6-311G(d,p) level was selected because it provides a reasonable balance between computational cost and the description of molecular geometries, electronic distributions, hydrogen bonding, and electrostatic-potential characteristics [62,63].

The optimized wavefunctions were analyzed using Multiwfn 3.8, and the ESP, IGMH, and IRI isosurfaces were visualized using VMD 1.9.4. The interaction energy was calculated as follows:

(3) $$∆E=E_{AB}-E_{A}-E_{B}$$

where *E*_AB_ is the lowest binding energy of the mixture, *E*_A_ is the energy of component A, and *E*_B_ is the energy of component B. The calculated interaction energies are presented in Table 2.

### 3.7. Statistical Analysis

All experiments were performed in triplicate, and the results are expressed as mean ± standard deviation. Statistical significance was evaluated by one-way ANOVA using OriginPro 2021, with *p* < 0.05 considered statistically significant.

## 4. Conclusions

In this study, a deep eutectic solvent composed of levulinic acid and ethylene glycol (molar ratio of 1:2) was successfully constructed and combined with pulsed ultrasound-assisted extraction technology to achieve efficient green extraction of curcumin. Under single-factor optimization conditions (liquid-solid ratio: 20 mL·g^−1^, ultrasonic power: 200 W, ultrasonication time: 55 min), the extracted amount of curcumin reached 99.9 mg·g^−1^, which was significantly higher than that reported for various solvents. Scanning electron microscopy revealed that ultrasound-assisted DES treatment significantly disrupted the cell structure of turmeric powder and promoted mass transfer. Quantum chemical calculations and molecular surface electrostatic potential analysis showed that there were strong hydrogen bonds and π-π interactions between the ALCA/EG system and curcumin, with van der Waals forces and hydrogen bonds as the main driving forces, and ALCA playing a leading role in binding. IGMH and IRI analysis further confirmed that the weak interaction strength of ALCA/EG on curcumin was significantly higher than that of demethoxycurcumin and bisdemethoxycurcumin, showing a certain selectivity. The ALCA/EG system enabled efficient curcuminoid extraction under mild conditions and provides a basis for further evaluation in natural-product extraction.

## Figures and Tables

**Table 1:** Extraction rate of curcumin compounds with different extractants.

No.	Extraction Solvent	Solvent Type	Extraction Method	CUR Amount (mg·g^−1^)
1	ChCl:Pyr	DES	Ultrasound-assisted	11.9
2	ALCA:n-BuoH	DES	Ultrasound-assisted	23.9
3	ALCA:Pyr	DES	Ultrasound-assisted	51.8
4	ChCl:OxA	DES	Ultrasound-assisted	54.3
5	ALCA:Cap	DES	Ultrasound-assisted	65.2
6	ALCA:OxA	DES	Ultrasound-assisted	66.9
7	ALCA:Gly	DES	Ultrasound-assisted	74.5
8	ALCA:LA	DES	Ultrasound-assisted	75.6
9	OA:DA	DES	Ultrasound-assisted	77.2
10	ALCA:EG	DES	Ultrasound-assisted	99.9

**Table 2:** Interaction energies of curcuminoid–DES complexes calculated by quantum chemical analysis.

Substance	E_AB_ (a.u)	ΔE (a.u)
ALCA-CUR	−1685.0816	−0.0457
EG-CUR	−1526.4521	−0.0367
LA-CUR	−1607.6331	−0.0135
Pyr-CUR	−1596.9436	−0.0238
Cap-CUR	−1729.0183	−0.0101
OxA-CUR	−1642.3604	−0.0229
ALCA-DMC	−1561.1650	−0.0352
EG-DMC	−1371.4031	−0.0373
LA-DMC	−1484.2057	−0.0148
Pyr-DMC	−1483.0293	−0.0287
Cap-DMC	−1604.5541	−0.0152
OxA-DMC	−1518.8898	−0.0263
ALCA-BDMC	−1447.2297	−0.0332
EG-BDMC	−1257.4917	−0.0325
LA-BDMC	−1370.2849	−0.0146
Pyr-BDMC	−1369.1205	−0.0158
Cap-BDMC	−1490.6430	−0.0296
OxA-BDMC	−1404.9800	−0.0247
ALCA-EG-CUR	−1915.4242	−0.0415
ALCA-EG-DMC	−1800.8733	−0.0267
ALCA-EG-BDMC	−1676.2444	−0.0112
ALCA-EG	−651.5036	−0.0323
ALCA-LA	−764.8430	−0.0260
ALCA-Pyr	−759.3645	−0.0128
ALCA-Cap	−880.8850	−0.0116
ALCA-OxA	−795.2231	−0.0159

## Data Availability

The numerical data from Figure 4 are listed in Tables S1–S5. The data supporting the findings of this study can be obtained from the corresponding author upon reasonable request.

## Supplementary Materials

The following supporting information can be downloaded at: https://www.mdpi.com/article/10.3390/molecules31162818/s1.
