# Supplementary material for: Ultrasound-Assisted Extraction of Curcumin Using a Deep Eutectic Solvent: Molecular Insights into the Extraction Mechanism
Source: Molecules. 2026 Aug 13;31(16):2818. doi: 10.3390/molecules31162818 (PMC13515862; doi:10.3390/molecules31162818)
Supplement: Supplementary file 1 [file molecules-31-02818-s001.zip › Supplementary Material.pdf]

Supporting Information

Ultrasound-Assisted Extraction of Curcumin Using a Deep Eutectic Solvent: Molecular Insights into the Extraction Mechanism

Ning Sun[a] | Xinyu Guo[a] | Enxi Li[a] | Yan Zhou[a] | Shuli Yin[a] | Peizhe Cui[a, *]

[a] *College of Chemical Engineering, Qingdao University of Science and Technology, Qingdao, 266042, People's Republic of China*

***Corresponding Author**

Peizhe Cui

College of Chemical Engineering

Qingdao University of Science and Technology

Qingdao, 266042, People's Republic of China

*Email: cpzmagic@qust.edu.cn (P. Cui)

Number of pages: 19

Number of formulas:0

Number of Tables: 6

Number of Figures: 12

Figure S1. Calibration curves of curcuminoid standards in methanol solution

Figure S2. High performance liquid chromatography of curcuminoid standards

Figure S3. FTIR spectra of ALCA, OxA, Cap, Gly, and n-BuOH

Figure S4. ^{1}H NMR spectra of ALCA, OxA, Cap, Gly, and n-BuOH.

Figure S5. The surface van der Waals electrostatic potential analysis diagram of CUR + ALCA and HBD

Figure S6. The surface van der Waals electrostatic potential analysis diagram of DMC + ALCA and HBD (red: positive potential; blue:negative potential).

Figure S7. Molecular electrostatic potential maps of BDMC–ALCA–HBD complexes (red: positive potential; blue:negative potential).

Figure S8. Colored comparison diagram of mapping function in IGMH.

Figure S9. IGMH analysis of interactions between ALCA and different hydrogen-bond donors: (a)ALCA-Pyr;(b) ALCA-OxA;(c) ALCA-Cap ;(d) ALCA-LA

Figure S10. IRI contour and color-filled maps of CUR–EG, DMC–EG, and BDMC–EG complexes

Figure S11. IRI contour and color-filled maps of CUR–ALCA, DMC–ALCA, and BDMC–ALCA complexes

Figure S12. IRI contour and color-filled maps of CUR–Pyr, DMC–Pyr, and BDMC–Pyr complexes

Table S1. Experimental and fitted parameters of Peleg kinetic model under different ultrasonic powers

Table S2. Experimental and fitted parameters of Peleg kinetic model for different HBA: HBD molar ratios

Table S3. Experimental and fitted parameters of Peleg kinetic model at different liquid-to-solid ratios

Table S4. Experimental and fitted parameters of Peleg kinetic model at different extraction times

Table S5. Extraction yields of curcuminoids using different deep eutectic solvents

Table S6. Abbreviations of compounds and analytical terms used in this study

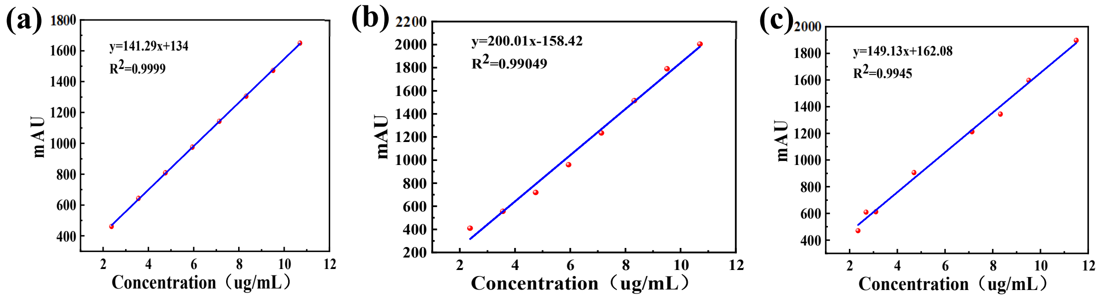

Figure S1. Calibration curves of curcuminoid standards in methanol solution

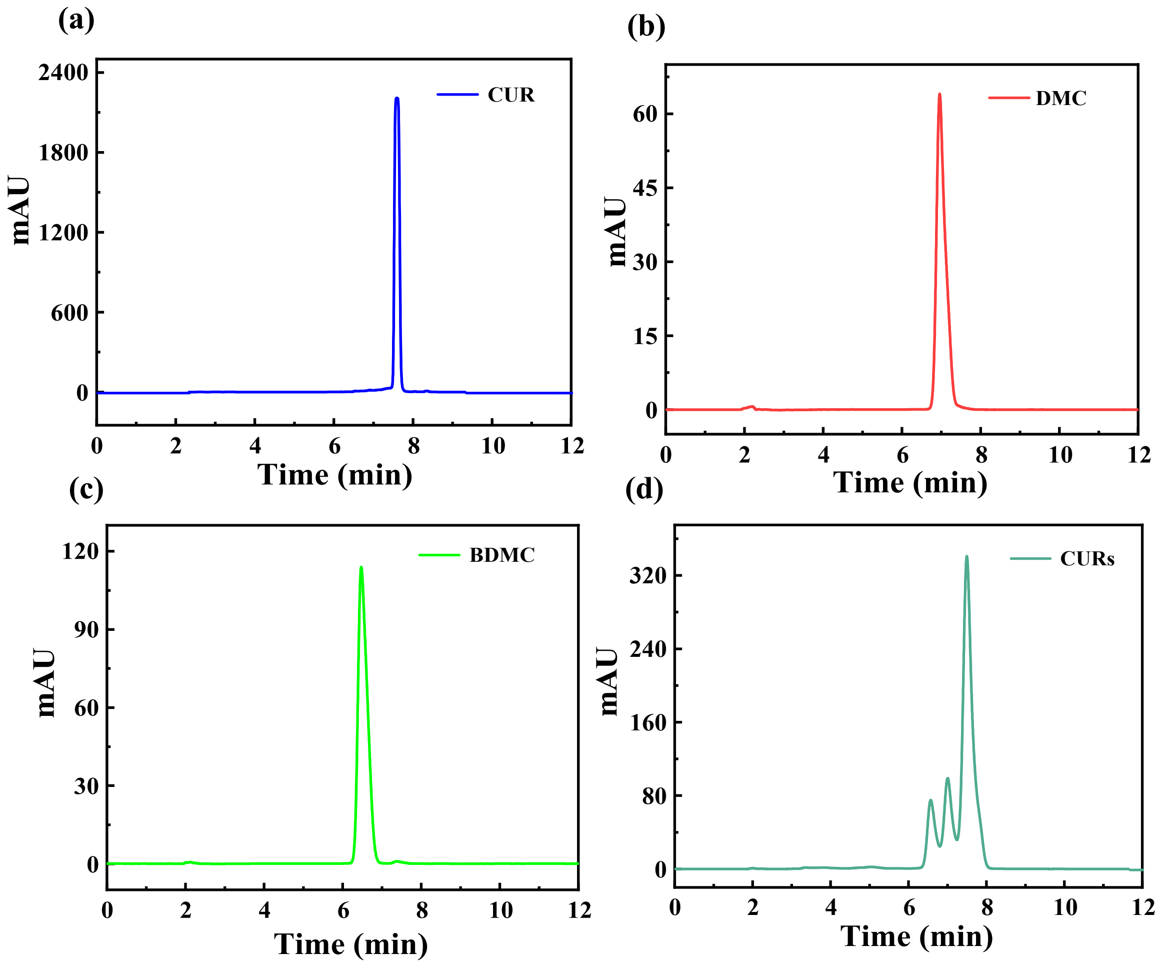

Figure S2. High performance liquid chromatography of curcuminoid standards

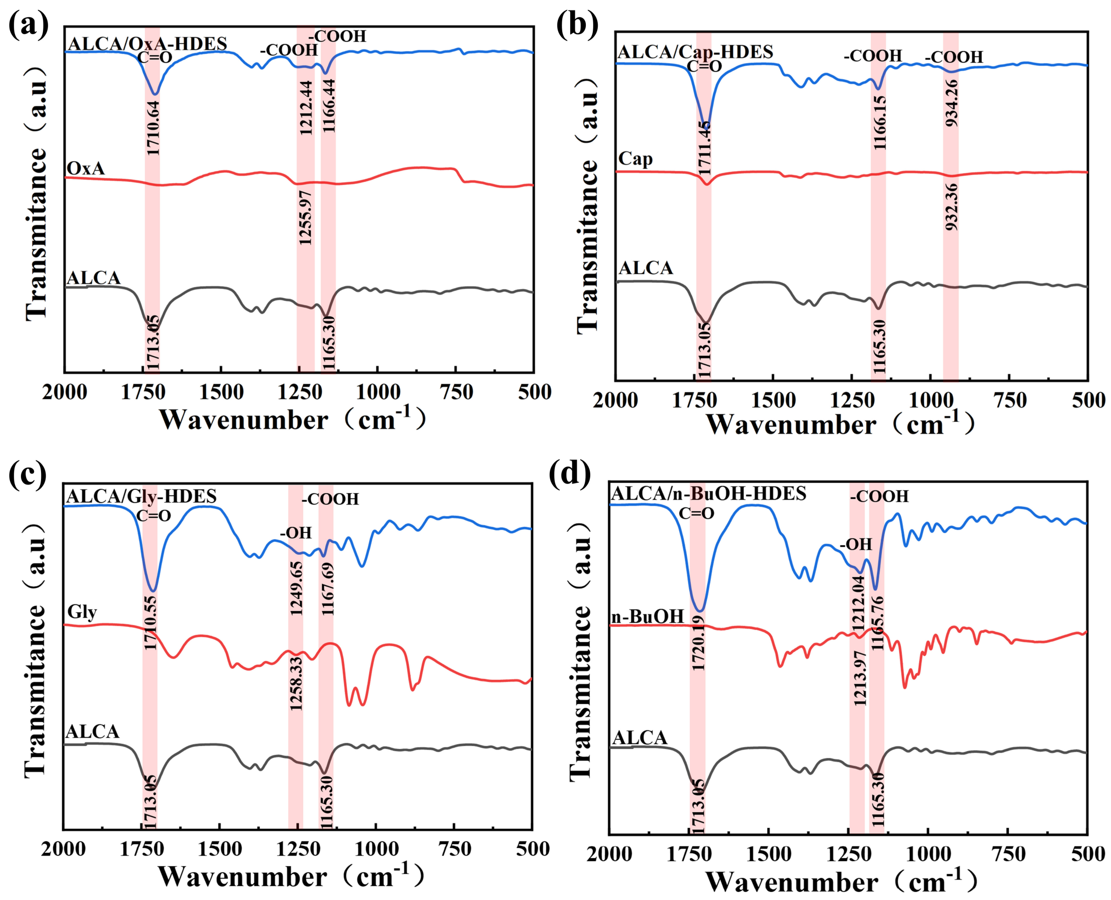

Figure S3. FTIR spectra of ALCA, OxA, Cap, Gly, and n-BuOH

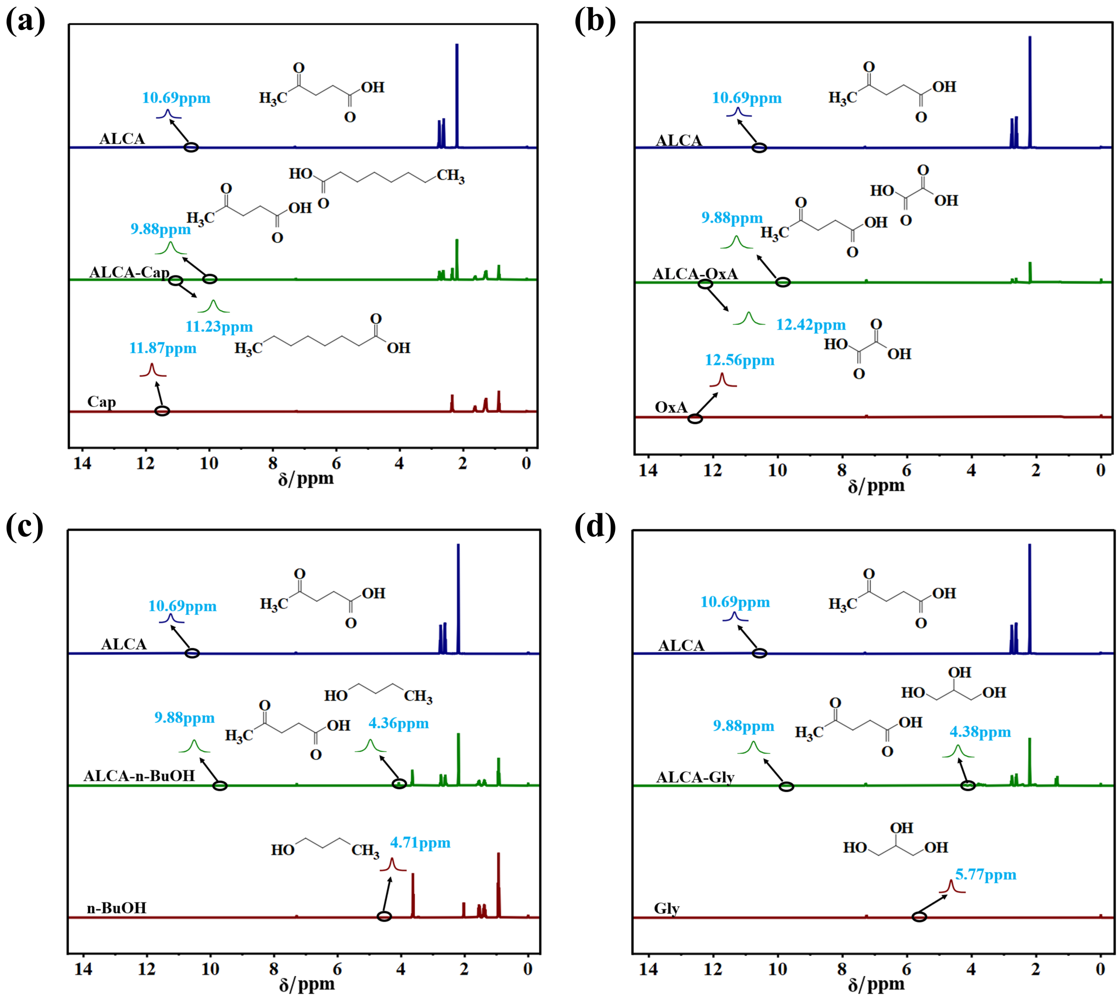

Figure S4. ^{1}H NMR spectra of ALCA, OxA, Cap, Gly, and n-BuOH.

Table S1. Experimental and fitted parameters of Peleg kinetic model under different ultrasonic powers

Power/w	Experimental C_{eq} (mg/g)	K_1 (min.g/g)	K_2 (g/mg)	Predicted C_{eq} (mg/g)	RMSE (mg/g)
100	84.49	0.1523	0.0255	85.01	0.46
200	97.29	0.0452	0.0154	98.32	0.25
300	88.83	0.0638	0.0123	89.10	0.39
400	88.7	0.0318	0.0145	89.21	0.71
500	87.51	0.0295	0.0126	88.06	0.53

Table S2. Experimental and fitted parameters of Peleg kinetic model for different HBA: HBD molar ratios

HBA：HBD	Experimental C_{eq} (mg/g)	K_1 (min.g/g)	K_2 (g/mg)	Predicted C_{eq} (mg/g)	RMSE (mg/g)
1:1	79.66	0.0964	0.0126	80.02	0.57
1:2	93.05	0.0384	0.0135	94.26	0.39
1:3	63.92	0.0125	0.0105	64.32	0.48
2:1	87.58	0.0421	0.0121	88.25	0.37
3:1	80.50	0.0187	0.0116	81.03	0.75

Table S3. Experimental and fitted parameters of Peleg kinetic model at different liquid-to-solid ratios

L：S	Experimental C_{eq} (mg/g)	K_1 (min.g/g)	K_2 (g/mg)	Predicted C_{eq} (mg/g)	RMSE (mg/g)
10	86.00	0.1501	0.0149	86.65	0.32
20	99.9	0.0154	0.0122	100.21	0.51
30	73.15	0.0428	0.0118	74.23	0.49
40	90.21	0.0726	0.0237	91.01	0.42
50	83.68	0.0525	0.0312	84.23	0.23

Table S4. Experimental and fitted parameters of Peleg kinetic model at different extraction times

t/min	Experimental C_{eq} (mg/g)	K_1 (min.g/g)	K_2 (g/mg)	Predicted C_{eq} (mg/g)	RMSE (mg/g)
25	79.86	0.2875	0.0195	80.21	0.37
35	83.5	0.0453	0.0287	84.03	0.45
45	78	0.0245	0.0115	78.56	0.36
55	96.1	0.0575	0.0116	96.85	0.53
65	86.63	0.0419	0.0135	87.35	0.28

Table S5. Extraction yields of curcuminoids using different deep eutectic solvents

No.	Extraction Solvent	Solvent Type	Extraction Method	CURs Amount (mg/g)
1	OA: DA	DES	Ultrasound-assisted	87.2
2	ChCl : OxA	DES	Ultrasound-assisted	54.3
3	ChCl:Pyr	DES	Ultrasound-assisted	11.9
4	ALCA: Gly	DES	Ultrasound-assisted	74.5
5	ALCA: n-BuoH	DES	Ultrasound-assisted	43.9
6	ALCA: EG	DES	Ultrasound-assisted	99.9
7	ALCA: Cap	DES	Ultrasound-assisted	65.2
8	ALCA: Pyr	DES	Ultrasound-assisted	51.8
9	ALCA: LA	DES	Ultrasound-assisted	75.6
10	ALCA: OxA	DES	Ultrasound-assisted	66.9

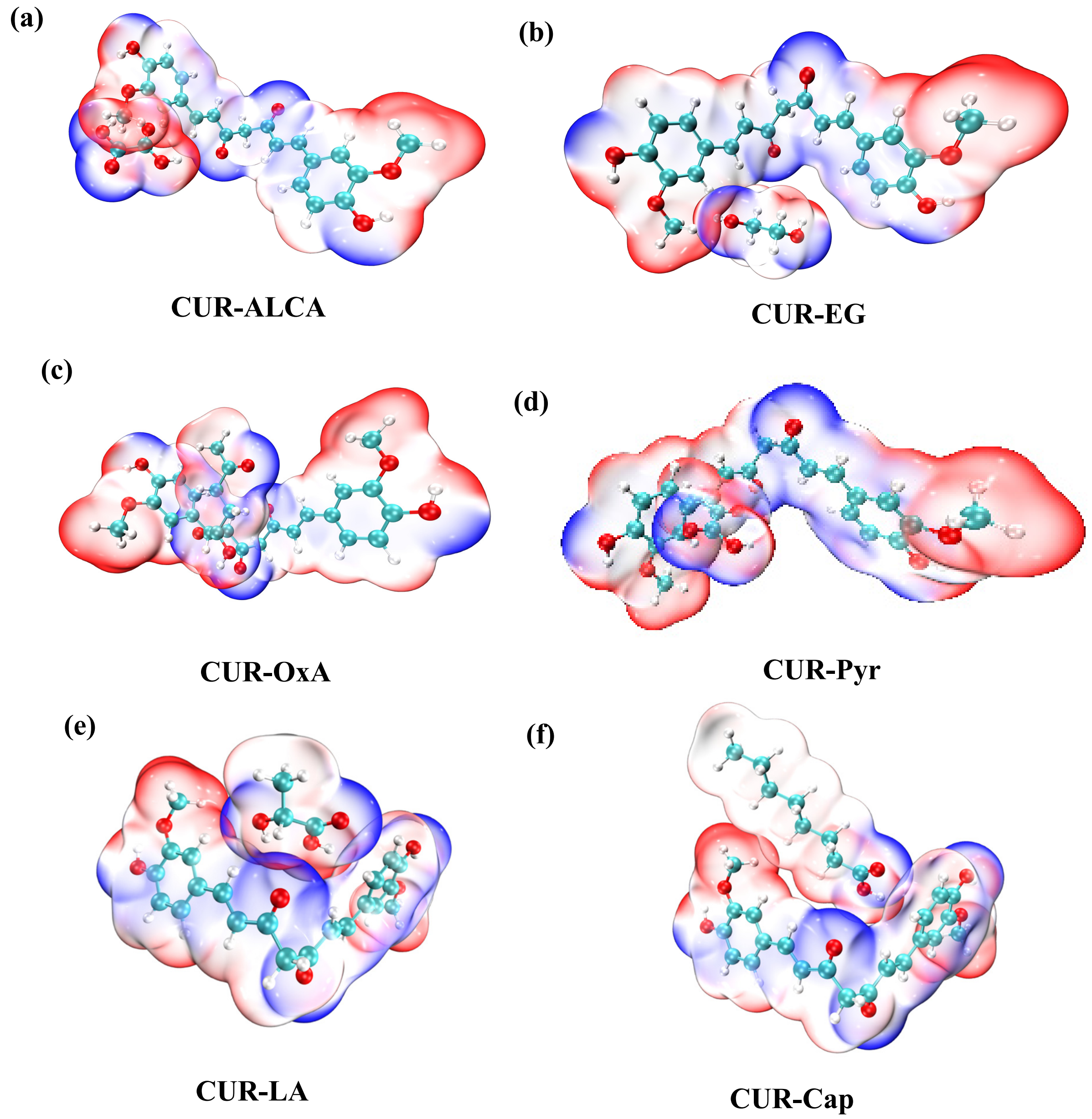

Figure S5. Molecular electrostatic potential maps of CUR–ALCA–HBD complexes

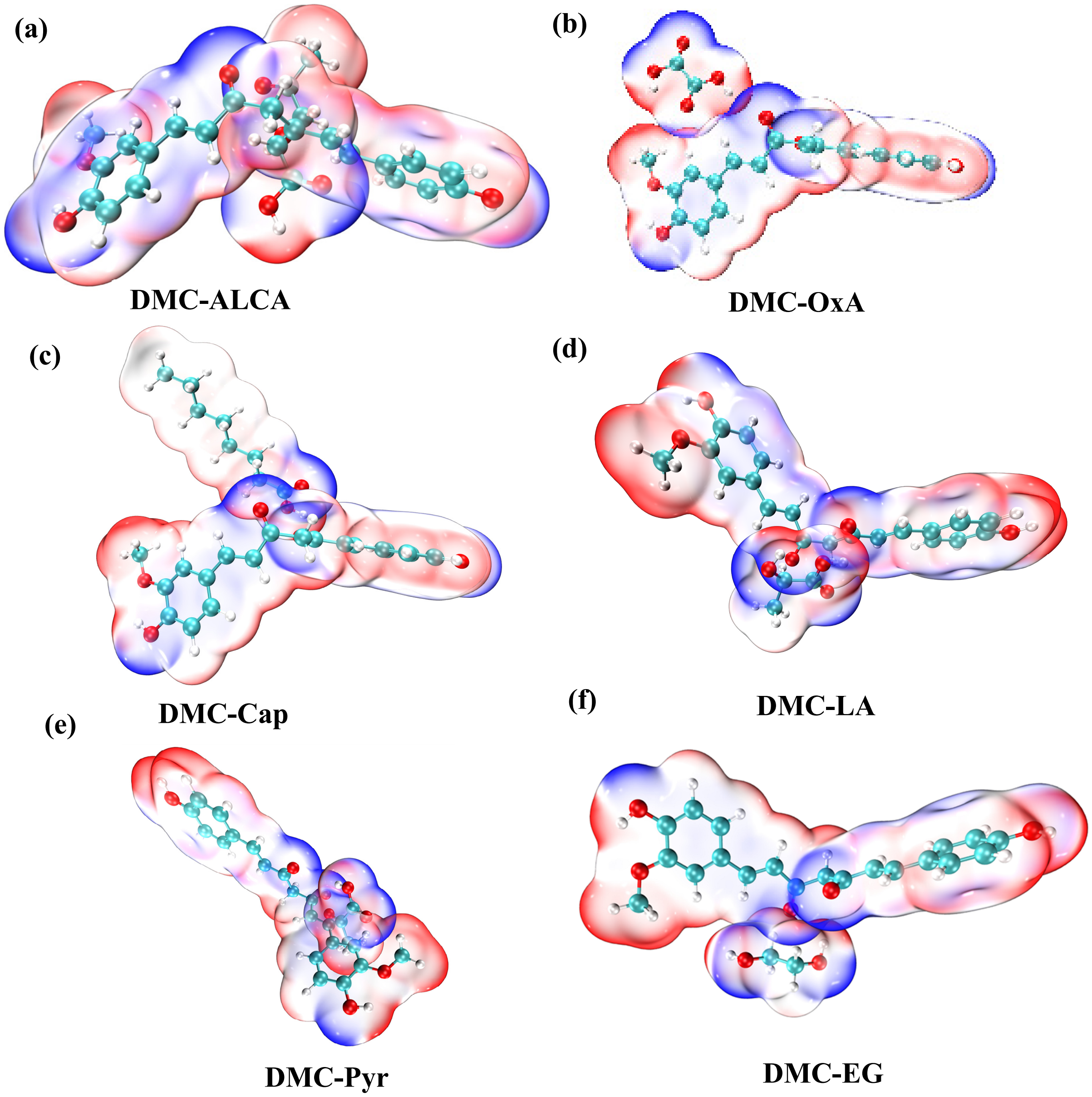

Figure S6. Molecular electrostatic potential maps of DMC–ALCA–HBD complexes (red: positive potential; blue:negative potential).

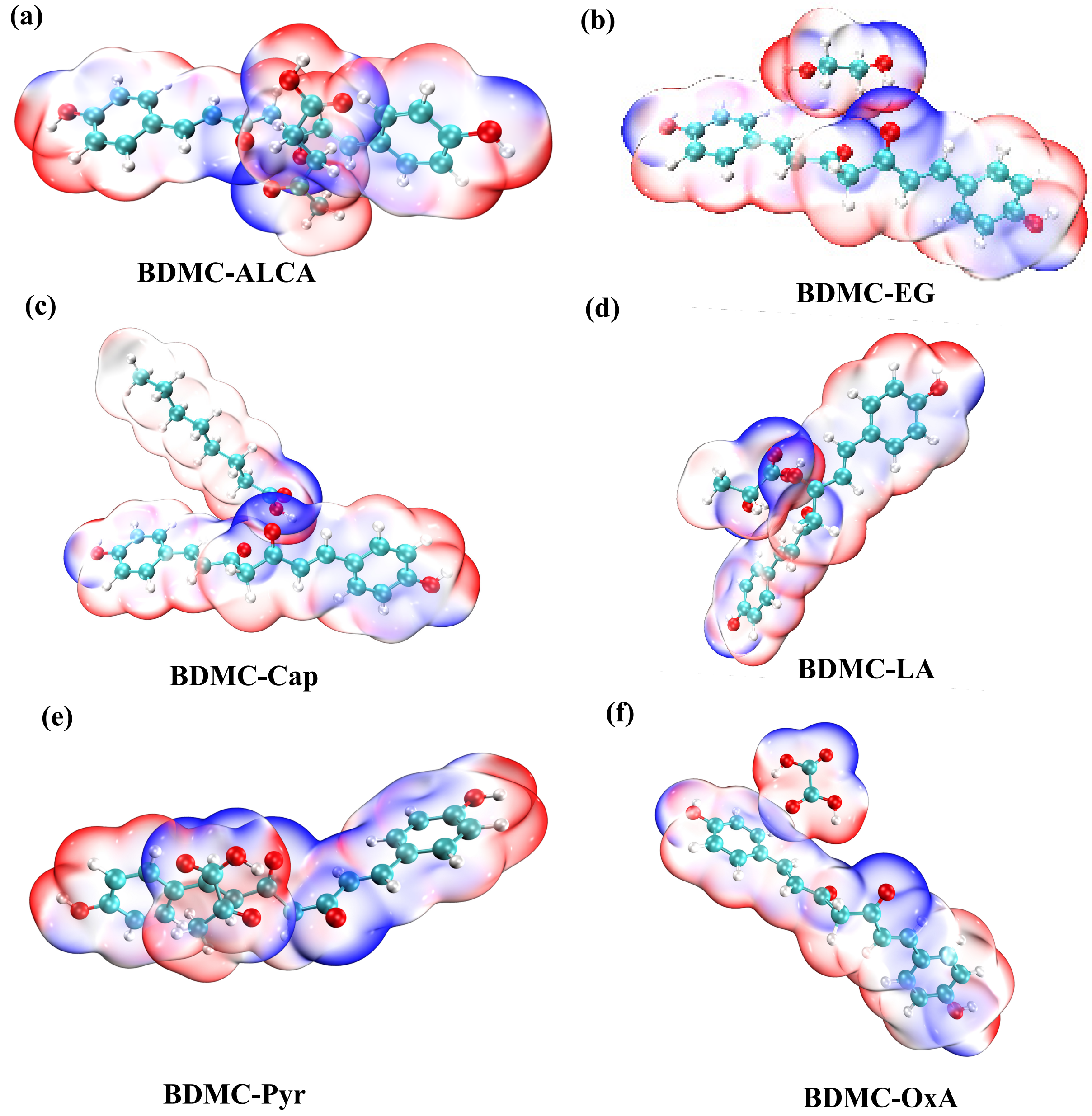

Figure S7. Molecular electrostatic potential maps of BDMC–ALCA–HBD complexes (red: positive potential; blue:negative potential).

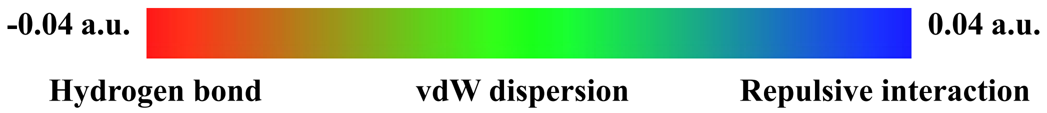

Figure S8. Color scale of interaction types in IGMH analysis

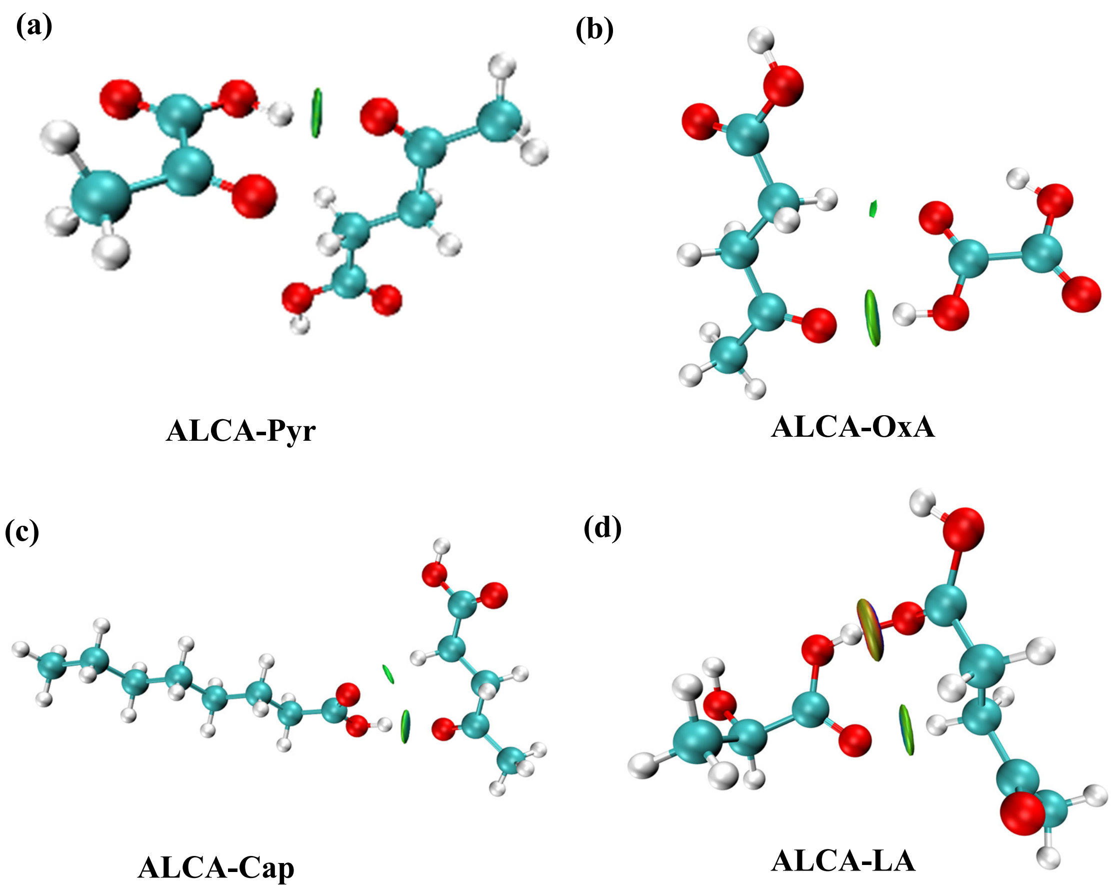

Figure S9. IGMH analysis of interactions between ALCA and different hydrogen-bond donors: (a)ALCA-Pyr;(b) ALCA-OxA;(c) ALCA-Cap ;(d) ALCA-LA

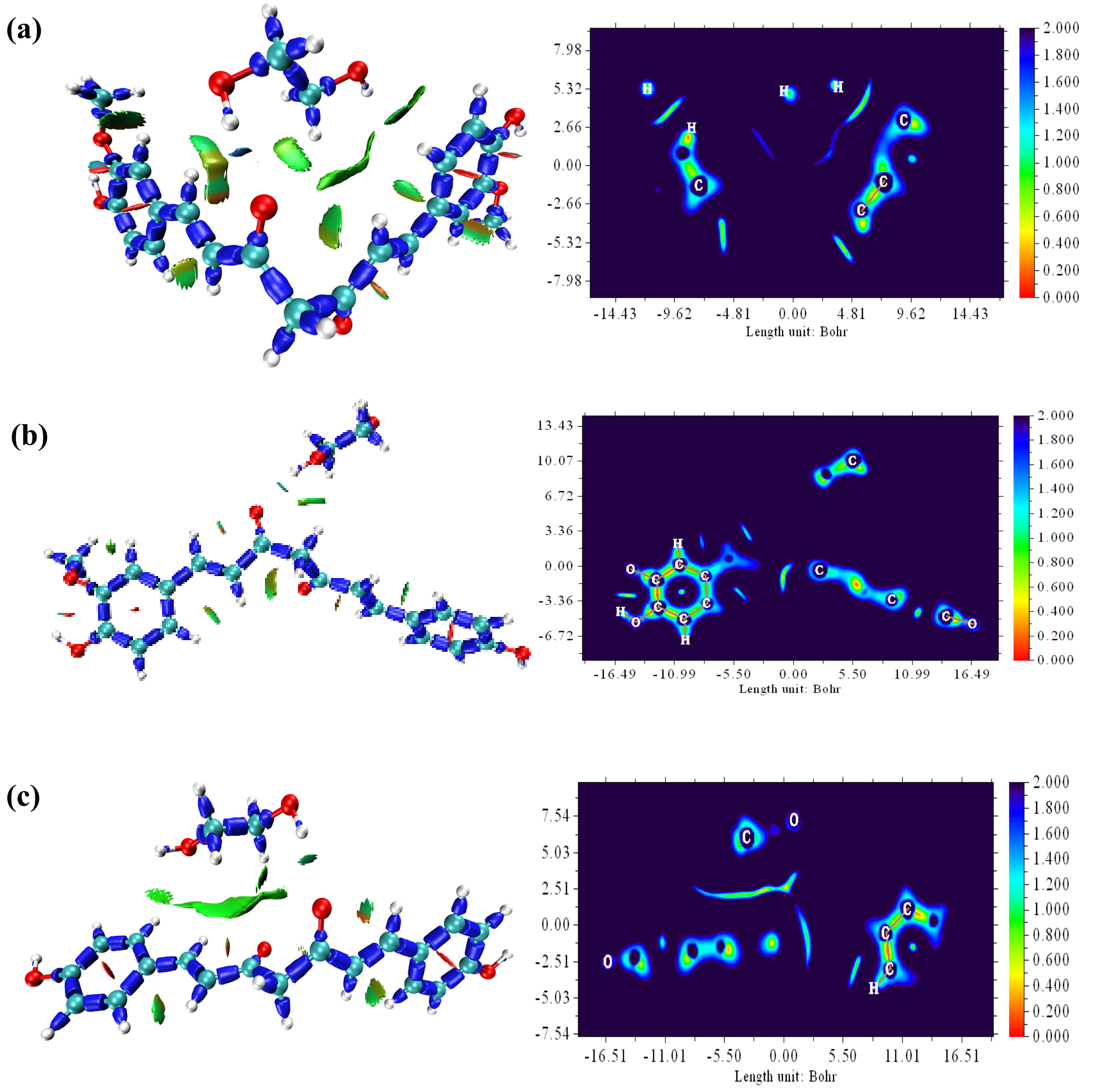

Figure S10. IRI contour and color-filled maps of CUR–EG, DMC–EG, and BDMC–EG complexes

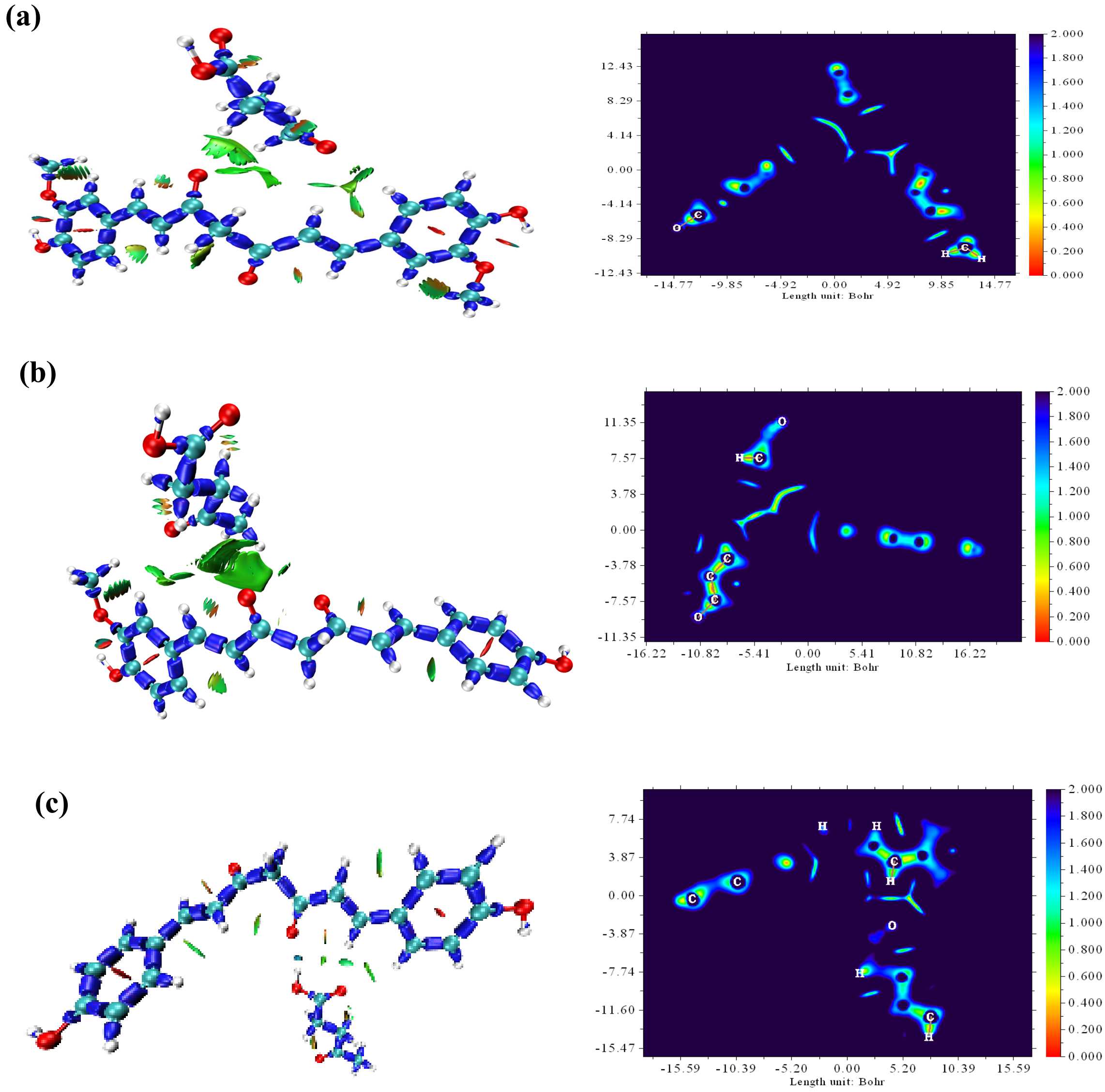

Figure S11. IRI contour and color-filled maps of CUR–ALCA, DMC–ALCA, and BDMC–ALCA complexes

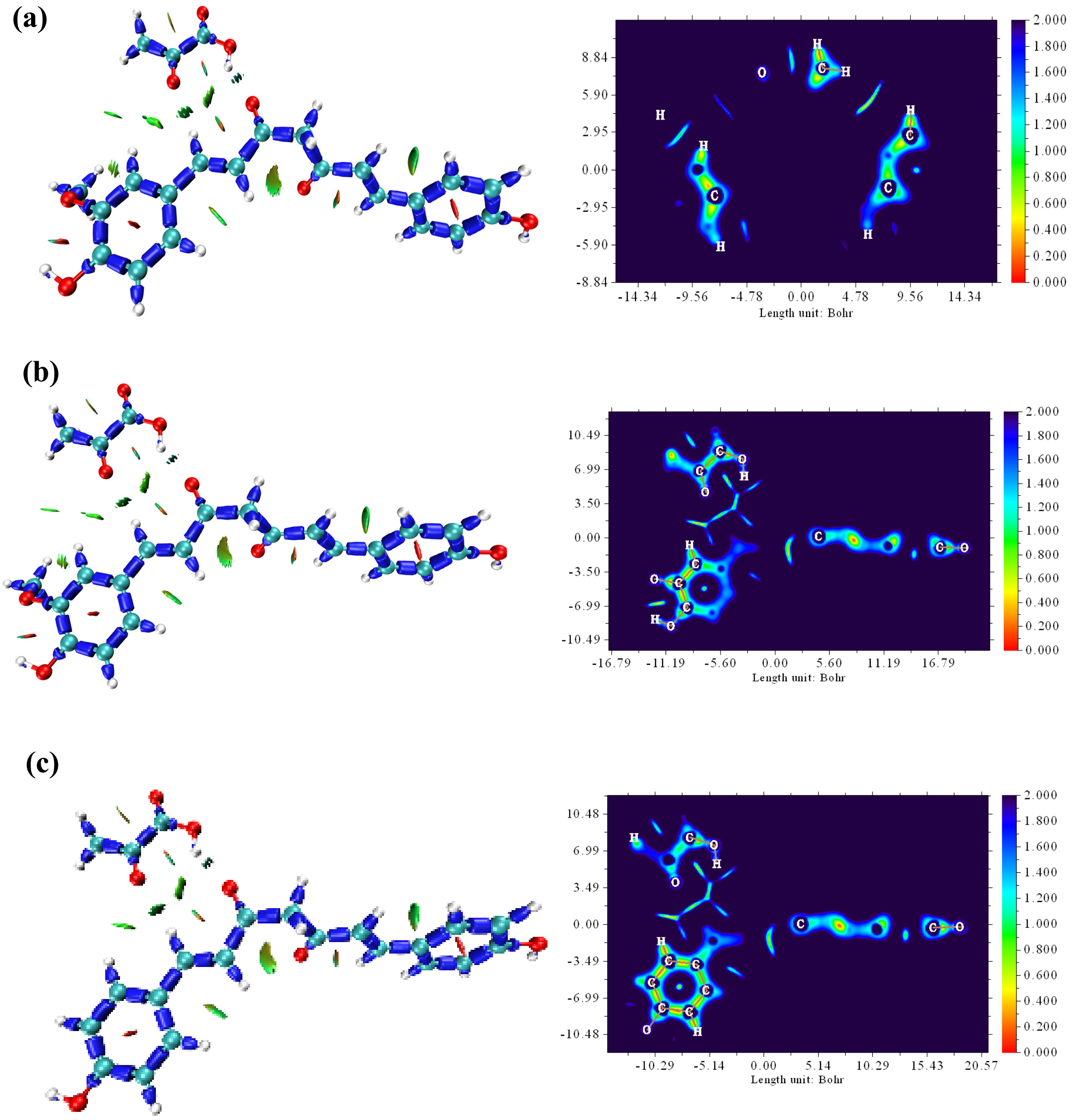

Figure S12. IRI contour and color-filled maps of CUR–Pyr, DMC–Pyr, and BDMC–Pyr complexes

Table S6. Abbreviations of compounds and analytical terms used in this study

Name	abbreviation
choline chloride	ChCl
Geraniol	Ger
L-proline	Pro
levulinic acid	ALCA
menthol	Men
dodecanoic acid	La
oleic acid	OA
capric acid	DA
pyruvic acid	Pyr
oxalic acid	OxA
glycol	EG
lactic acid	LA
caprylic acid	Cap
glycerol	Gly
n-butyl alcohol	n-BuOH
deep eutectic solvent	DES
curcumin	CUR
matrdemethoxycurcumin	DMC
bisdemethoxycurcumin	BDMC
hydrogen bond acceptor	HBA
hydrogen bond donor	HBD
high-performance liquid chromatography	HPLC
Fourier-transform infrared spectroscopy	FTIR
proton nuclear magnetic resonance spectroscopy	^{1}H NMR
scanning electron microscopy	SEM
interaction region indicator	IRI
